# Supplementary material for: Pandemic-induced changes in household-level food diversity and diet quality in the U.S
Source: PLoS One. 2024 May 31;19(5):e0300839. doi: 10.1371/journal.pone.0300839 (PMC11142597; doi:10.1371/journal.pone.0300839)
Supplement: S1 Table — (DOCX) [file pone.0300839.s001.docx]

**S1 Table. Results for Berry Index and USDAScore regressions**

|  |  | Dependent variable: Natural log from monthly aggregation of household purchases | | |  |  |
| --- | --- | --- | --- | --- | --- | --- |
| Independent variable | Relative month |  | (1) | (2) |  |  |
|  |  |  | 24-category Berry Index | USDAScore1 |  |  |
| 1.pandemicyear#1.refmonth | -6 |  | -0.00920*** | -0.00238 |  |  |
|  |  |  | (0.003) | (0.003) |  |  |
| 1.pandemicyear#2.refmonth | -5 |  | -0.00525 | -0.00523* |  |  |
|  |  |  | (0.003) | (0.003) |  |  |
| 1.pandemicyear#3.refmonth | -4 |  | -0.00370 | 0.00304 |  |  |
|  |  |  | (0.003) | (0.003) |  |  |
| 1.pandemicyear#4.refmonth | -3 |  | -0.00483 | -0.00821*** |  |  |
|  |  |  | (0.003) | (0.003) |  |  |
| 1.pandemicyear#5.refmonth | -2 |  | -0.00506* | -0.00331 |  |  |
|  |  |  | (0.003) | (0.003) |  |  |
| 1.pandemicyear#6.refmonth | -1 |  | (omitted) | |  |  |
|  |  |  |  |  |  |  |
| 1.pandemicyear#7.refmonth | 0 |  | 0.0256*** | 0.0763*** |  |  |
|  |  |  | (0.003) | (0.003) |  |  |
| 1.pandemicyear#8.refmonth | 1 |  | 0.0136*** | 0.0656*** |  |  |
|  |  |  | (0.003) | (0.003) |  |  |
| 1.pandemicyear#9.refmonth | 2 |  | 0.0220*** | 0.0846*** |  |  |
|  |  |  | (0.004) | (0.003) |  |  |
| 1.pandemicyear#10.refmonth | 3 |  | 0.0107*** | 0.0551*** |  |  |
|  |  |  | (0.003) | (0.003) |  |  |
| 1.pandemicyear#11.refmonth | 4 |  | 0.0123*** | 0.0388*** |  |  |
|  |  |  | (0.003) | (0.003) |  |  |
| 1.pandemicyear#12.refmonth | 5 |  | 0.0129*** | 0.0437*** |  |  |
|  |  |  | (0.003) | (0.003) |  |  |
| 1.pandemicyear#13.refmonth | 6 |  | 0.0137*** | 0.0305*** |  |  |
|  |  |  | (0.003) | (0.003) |  |  |
| 1.pandemicyear |  |  | -0.00312 | -0.0143*** |  |  |
|  |  |  | (0.002) | (0.002) |  |  |
| 1.refmonth |  |  | 0.000644 | -0.0218*** |  |  |
|  |  |  | (0.002) | (0.002) |  |  |
| 2.refmonth |  |  | 0.00450** | -0.00839*** |  |  |
|  |  |  | (0.002) | (0.002) |  |  |
| 3.refmonth |  |  | 0.0124*** | 0.0103*** |  |  |
|  |  |  | (0.002) | (0.002) |  |  |
| 4.refmonth |  |  | 0.00717*** | -0.00242 |  |  |
|  |  |  | (0.002) | (0.002) |  |  |
| 5.refmonth |  |  | 0.00840*** | 0.0167*** |  |  |
|  |  |  | (0.002) | (0.002) |  |  |
| 6.refmonth |  |  | (omitted) | |  |  |
|  |  |  |  |  |  |  |
| 7.refmonth |  |  | -0.00125 | -0.00378* |  |  |
|  |  |  | (0.002) | (0.002) |  |  |
| 8.refmonth |  |  | -0.00149 | -0.00885*** |  |  |
|  |  |  | (0.002) | (0.002) |  |  |
| 9.refmonth |  |  | -0.00888*** | -0.0240*** |  |  |
|  |  |  | (0.002) | (0.002) |  |  |
| 10.refmonth |  |  | -0.00941*** | -0.0197*** |  |  |
|  |  |  | (0.002) | (0.002) |  |  |
| 11.refmonth |  |  | -0.00788*** | -0.0105*** |  |  |
|  |  |  | (0.002) | (0.002) |  |  |
| 12.refmonth |  |  | -0.0138*** | -0.0349*** |  |  |
|  |  |  | (0.002) | (0.003) |  |  |
| 13.refmonth |  |  | -0.0117*** | -0.0360*** |  |  |
|  |  |  | (0.002) | (0.002) |  |  |
| _cons |  |  | 4.393*** | 1.769*** |  |  |
|  |  |  | (0.002) | (0.003) |  |  |
| N |  |  | 1075123 | 1066938 |  |  |
| Significance levels: * 10 percent, ** 5 percent, *** 1 percent. Standard errors (in parentheses) are clustered by county. Only estimated coefficients (and their standard errors) for interaction terms are used for the event-study plots. | | | | | |  |
