# Supplementary material for: Pandemic-induced changes in household-level food diversity and diet quality in the U.S
Source: PLoS One. 2024 May 31;19(5):e0300839. doi: 10.1371/journal.pone.0300839 (PMC11142597; doi:10.1371/journal.pone.0300839)
Supplement: S1 Appendix — (DOCX) [file pone.0300839.s002.docx]

**S2 Appendix. Results for Berry Index regressions with heterogeneity.**

Results for Fig 2: Berry Index by Census region of residence

|  |  | Dependent variable: Natural log of Berry Index | | | | |
| --- | --- | --- | --- | --- | --- | --- |
| Independent variable | Relative month | (1) | (2) | (3) | (4) |  |
|  |  | Northeast | Midwest | South | West |  |
| 1.pandemicyear#1.refmonth | -6 | -0.00837* | -0.000326 | -0.0157** | -0.00914 |  |
|  |  | (0.005) | (0.007) | (0.006) | (0.007) |  |
| 1.pandemicyear#2.refmonth | -5 | -0.00239 | 0.00779 | -0.0131** | -0.00998** |  |
|  |  | (0.005) | (0.007) | (0.006) | (0.005) |  |
| 1.pandemicyear#3.refmonth | -4 | -0.00385 | 0.00363 | -0.00608 | -0.00869 |  |
|  |  | (0.004) | (0.006) | (0.006) | (0.006) |  |
| 1.pandemicyear#4.refmonth | -3 | 0.00392 | 0.00496 | -0.0140** | -0.00783 |  |
|  |  | (0.006) | (0.007) | (0.006) | (0.005) |  |
| 1.pandemicyear#5.refmonth | -2 | -0.00315 | 0.00830 | -0.0129** | -0.00925 |  |
|  |  | (0.005) | (0.006) | (0.006) | (0.006) |  |
| 1.pandemicyear#6.refmonth | -1 | (omitted) | | | | |
|  |  |  |  |  |  |  |
| 1.pandemicyear#7.refmonth | 0 | 0.0231*** | 0.0338*** | 0.0243*** | 0.0197*** |  |
|  |  | (0.005) | (0.006) | (0.006) | (0.007) |  |
| 1.pandemicyear#8.refmonth | 1 | 0.000748 | 0.0242*** | 0.0118* | 0.0147** |  |
|  |  | (0.006) | (0.007) | (0.006) | (0.007) |  |
| 1.pandemicyear#9.refmonth | 2 | 0.0109* | 0.0353*** | 0.0197*** | 0.0189*** |  |
|  |  | (0.006) | (0.009) | (0.006) | (0.007) |  |
| 1.pandemicyear#10.refmonth | 3 | 0.0113** | 0.0175** | 0.00468 | 0.0131** |  |
|  |  | (0.006) | (0.007) | (0.006) | (0.006) |  |
| 1.pandemicyear#11.refmonth | 4 | 0.0134*** | 0.0196*** | 0.00619 | 0.0136** |  |
|  |  | (0.005) | (0.007) | (0.007) | (0.006) |  |
| 1.pandemicyear#12.refmonth | 5 | 0.0150* | 0.0244*** | 0.00549 | 0.0100 |  |
|  |  | (0.008) | (0.007) | (0.006) | (0.006) |  |
| 1.pandemicyear#13.refmonth | 6 | 0.0112* | 0.0136** | 0.00912 | 0.0249*** |  |
|  |  | (0.006) | (0.007) | (0.006) | (0.005) |  |
| 1.pandemicyear |  | -0.00244 | -0.0144*** | 0.00308 | -0.000794 |  |
|  |  | (0.002) | (0.006) | (0.005) | (0.003) |  |
| 1.refmonth |  | -0.00551 | 0.000162 | 0.00764* | -0.00683 |  |
|  |  | (0.004) | (0.004) | (0.004) | (0.005) |  |
| 2.refmonth |  | -0.00426 | 0.000777 | 0.0131*** | 0.000643 |  |
|  |  | (0.005) | (0.004) | (0.004) | (0.004) |  |
| 3.refmonth |  | 0.00607* | 0.0126*** | 0.0161*** | 0.0104*** |  |
|  |  | (0.003) | (0.004) | (0.005) | (0.004) |  |
| 4.refmonth |  | -0.00512 | 0.00526 | 0.0150*** | 0.00543 |  |
|  |  | (0.006) | (0.004) | (0.004) | (0.004) |  |
| 5.refmonth |  | 0.00541 | 0.00689* | 0.0130*** | 0.00417 |  |
|  |  | (0.004) | (0.004) | (0.004) | (0.004) |  |
| 6.refmonth |  | (omitted) | | | | |
|  |  |  |  |  |  |  |
| 7.refmonth |  | -0.00274 | -0.00342 | 0.00160 | -0.00256 |  |
|  |  | (0.004) | (0.004) | (0.004) | (0.004) |  |
| 8.refmonth |  | -0.00248 | -0.00421 | 0.00619 | -0.0119** |  |
|  |  | (0.004) | (0.004) | (0.004) | (0.005) |  |
| 9.refmonth |  | -0.00943** | -0.0132*** | -0.00335 | -0.0134*** |  |
|  |  | (0.004) | (0.005) | (0.004) | (0.005) |  |
| 10.refmonth |  | -0.0151*** | -0.0108*** | -0.00488 | -0.0114*** |  |
|  |  | (0.003) | (0.004) | (0.004) | (0.004) |  |
| 11.refmonth |  | -0.00927** | -0.0126*** | -0.00253 | -0.0107** |  |
|  |  | (0.004) | (0.004) | (0.005) | (0.005) |  |
| 12.refmonth |  | -0.0248*** | -0.0199*** | -0.00476 | -0.0134*** |  |
|  |  | (0.007) | (0.004) | (0.004) | (0.004) |  |
| 13.refmonth |  | -0.0189*** | -0.00975*** | -0.00402 | -0.0230*** |  |
|  |  | (0.005) | (0.004) | (0.005) | (0.004) |  |
| _cons |  | 4.405*** | 4.397*** | 4.385*** | 4.393*** |  |
|  |  | (0.003) | (0.003) | (0.004) | (0.004) |  |
| N |  | 186295 | 276626 | 404562 | 207640 |  |
| Significance levels: * 10 percent, ** 5 percent, *** 1 percent. Standard errors (in parentheses) are clustered by county. Only estimated coefficients (and their standard errors) for interaction terms are used for the event-study plots. | | | | | | |

Results for Fig 2: Berry Index by age of children

|  |  | Dependent variable: Natural log of Berry Index | | | | |
| --- | --- | --- | --- | --- | --- | --- |
| Independent variable | Relative month | (1) | (2) | (3) | (4) |  |
|  |  | No children under 18 years old | Any young children | School-age children | Only middle-school or high-school children |  |
| 1.pandemicyear#1.refmonth | -6 | -0.00960*** | -0.0154 | -0.00458 | -0.00541 |  |
|  |  | (0.004) | (0.012) | (0.015) | (0.012) |  |
| 1.pandemicyear#2.refmonth | -5 | -0.00578 | -0.0185* | -0.00599 | 0.0118 |  |
|  |  | (0.004) | (0.011) | (0.013) | (0.010) |  |
| 1.pandemicyear#3.refmonth | -4 | -0.00486 | 0.000382 | -0.00123 | 0.00675 |  |
|  |  | (0.003) | (0.008) | (0.011) | (0.011) |  |
| 1.pandemicyear#4.refmonth | -3 | -0.00563 | 0.00761 | -0.0127 | 0.00774 |  |
|  |  | (0.003) | (0.012) | (0.013) | (0.013) |  |
| 1.pandemicyear#5.refmonth | -2 | -0.00556* | -0.0118 | -0.00165 | 0.00218 |  |
|  |  | (0.003) | (0.012) | (0.010) | (0.012) |  |
| 1.pandemicyear#6.refmonth | -1 | (omitted) | | | | |
|  |  |  |  |  |  |  |
| 1.pandemicyear#7.refmonth | 0 | 0.0241*** | 0.0201*** | 0.0361*** | 0.0379*** |  |
|  |  | (0.004) | (0.007) | (0.011) | (0.010) |  |
| 1.pandemicyear#8.refmonth | 1 | 0.0117*** | 0.0141* | 0.0281** | 0.0221 |  |
|  |  | (0.004) | (0.008) | (0.012) | (0.017) |  |
| 1.pandemicyear#9.refmonth | 2 | 0.0238*** | 0.000406 | 0.0196 | 0.0141 |  |
|  |  | (0.004) | (0.015) | (0.015) | (0.012) |  |
| 1.pandemicyear#10.refmonth | 3 | 0.0121*** | 0.00385 | -0.0108 | 0.0229** |  |
|  |  | (0.004) | (0.012) | (0.016) | (0.011) |  |
| 1.pandemicyear#11.refmonth | 4 | 0.0125*** | 0.0152 | 0.00601 | 0.0157 |  |
|  |  | (0.004) | (0.013) | (0.013) | (0.011) |  |
| 1.pandemicyear#12.refmonth | 5 | 0.0108*** | 0.0120* | 0.0302* | 0.0202* |  |
|  |  | (0.004) | (0.007) | (0.017) | (0.010) |  |
| 1.pandemicyear#13.refmonth | 6 | 0.0136*** | 0.00857 | 0.0176* | 0.0139 |  |
|  |  | (0.004) | (0.012) | (0.010) | (0.013) |  |
| 1.pandemicyear |  | -0.00252 | 0.000333 | -0.00682 | -0.00926 |  |
|  |  | (0.003) | (0.005) | (0.008) | (0.010) |  |
| 1.refmonth |  | 0.00182 | -0.00474 | -0.0102 | 0.00135 |  |
|  |  | (0.002) | (0.004) | (0.011) | (0.004) |  |
| 2.refmonth |  | 0.00496* | 0.00144 | 0.00234 | 0.00285 |  |
|  |  | (0.003) | (0.005) | (0.008) | (0.004) |  |
| 3.refmonth |  | 0.0128*** | -0.000869 | 0.0146** | 0.0126*** |  |
|  |  | (0.002) | (0.005) | (0.006) | (0.004) |  |
| 4.refmonth |  | 0.00872*** | -0.00793 | 0.00383 | -0.000255 |  |
|  |  | (0.002) | (0.010) | (0.008) | (0.008) |  |
| 5.refmonth |  | 0.00817*** | 0.00510 | 0.0125** | 0.00860** |  |
|  |  | (0.002) | (0.005) | (0.006) | (0.004) |  |
| 6.refmonth |  | (omitted) | | | | |
|  |  |  |  |  |  |  |
| 7.refmonth |  | -0.000205 | -0.00385 | -0.00883 | -0.00461 |  |
|  |  | (0.003) | (0.005) | (0.008) | (0.004) |  |
| 8.refmonth |  | -0.000195 | -0.00530 | -0.00682 | -0.0103 |  |
|  |  | (0.003) | (0.005) | (0.008) | (0.007) |  |
| 9.refmonth |  | -0.00876*** | -0.0116** | -0.0138 | -0.00258 |  |
|  |  | (0.003) | (0.005) | (0.010) | (0.004) |  |
| 10.refmonth |  | -0.00894*** | -0.0170*** | -0.00751 | -0.0133*** |  |
|  |  | (0.002) | (0.005) | (0.006) | (0.004) |  |
| 11.refmonth |  | -0.00624** | -0.0214*** | -0.0154* | -0.0124*** |  |
|  |  | (0.003) | (0.006) | (0.008) | (0.004) |  |
| 12.refmonth |  | -0.0120*** | -0.0170*** | -0.0357*** | -0.00936** |  |
|  |  | (0.003) | (0.005) | (0.014) | (0.004) |  |
| 13.refmonth |  | -0.0115*** | -0.0171 | -0.0145* | -0.00879** |  |
|  |  | (0.003) | (0.011) | (0.008) | (0.004) |  |
| _cons |  | 4.391*** | 4.408*** | 4.405*** | 4.406*** |  |
|  |  | (0.002) | (0.004) | (0.006) | (0.004) |  |
| N |  | 889876 | 42101 | 79327 | 63819 |  |
| Significance levels: * 10 percent, ** 5 percent, *** 1 percent. Standard errors (in parentheses) are clustered by county. Only estimated coefficients (and their standard errors) for interaction terms are used for the event-study plots. | | | | | | |

Results for Fig 2: Berry Index by household income level

|  |  | Dependent variable: Natural log of Berry Index | | | | |
| --- | --- | --- | --- | --- | --- | --- |
| Independent variable | Relative month | (1) | (2) | (3) | (4) |  |
|  |  | Low income | Low-Medium income | Medium-High income | High income |  |
| 1.pandemicyear#1.refmonth | -6 | 0.00255 | -0.00109 | -0.0142*** | -0.0224*** |  |
|  |  | (0.008) | (0.007) | (0.005) | (0.007) |  |
| 1.pandemicyear#2.refmonth | -5 | 0.0110 | -0.00331 | -0.00679 | -0.0182** |  |
|  |  | (0.008) | (0.006) | (0.005) | (0.008) |  |
| 1.pandemicyear#3.refmonth | -4 | -0.0000718 | 0.00275 | -0.00788* | -0.00955 |  |
|  |  | (0.008) | (0.006) | (0.004) | (0.007) |  |
| 1.pandemicyear#4.refmonth | -3 | -0.000593 | -0.00461 | -0.00429 | -0.00909 |  |
|  |  | (0.007) | (0.006) | (0.005) | (0.008) |  |
| 1.pandemicyear#5.refmonth | -2 | 0.00594 | -0.000251 | -0.00920** | -0.0144** |  |
|  |  | (0.008) | (0.006) | (0.005) | (0.007) |  |
| 1.pandemicyear#6.refmonth | -1 | (omitted) | | | | |
|  |  |  |  |  |  |  |
| 1.pandemicyear#7.refmonth | 0 | 0.0201*** | 0.0235*** | 0.0250*** | 0.0337*** |  |
|  |  | (0.008) | (0.006) | (0.005) | (0.007) |  |
| 1.pandemicyear#8.refmonth | 1 | 0.0156* | 0.0166*** | 0.00519 | 0.0195** |  |
|  |  | (0.009) | (0.006) | (0.007) | (0.008) |  |
| 1.pandemicyear#9.refmonth | 2 | 0.0271*** | 0.0258*** | 0.0175*** | 0.0191*** |  |
|  |  | (0.009) | (0.006) | (0.007) | (0.007) |  |
| 1.pandemicyear#10.refmonth | 3 | 0.0190** | 0.0117* | 0.00969* | 0.00463 |  |
|  |  | (0.008) | (0.007) | (0.006) | (0.008) |  |
| 1.pandemicyear#11.refmonth | 4 | 0.00858 | 0.0190*** | 0.00953* | 0.00999 |  |
|  |  | (0.009) | (0.006) | (0.006) | (0.008) |  |
| 1.pandemicyear#12.refmonth | 5 | 0.0000161 | 0.0141** | 0.0202*** | 0.0111* |  |
|  |  | (0.010) | (0.006) | (0.006) | (0.007) |  |
| 1.pandemicyear#13.refmonth | 6 | 0.0195** | 0.0208*** | 0.00569 | 0.0104 |  |
|  |  | (0.008) | (0.006) | (0.006) | (0.008) |  |
| 1.pandemicyear |  | -0.0108* | -0.00780 | 0.00269 | 0.00121 |  |
|  |  | (0.006) | (0.005) | (0.004) | (0.005) |  |
| 1.refmonth |  | -0.00470 | -0.00184 | 0.000417 | 0.00839* |  |
|  |  | (0.005) | (0.004) | (0.004) | (0.004) |  |
| 2.refmonth |  | -0.00446 | 0.00976*** | 0.00411 | 0.00474 |  |
|  |  | (0.005) | (0.004) | (0.004) | (0.005) |  |
| 3.refmonth |  | 0.00606 | 0.00950** | 0.0161*** | 0.0160*** |  |
|  |  | (0.004) | (0.004) | (0.003) | (0.005) |  |
| 4.refmonth |  | 0.00469 | 0.0105*** | 0.00676* | 0.00513 |  |
|  |  | (0.004) | (0.004) | (0.004) | (0.006) |  |
| 5.refmonth |  | -0.00123 | 0.00886** | 0.0114*** | 0.0111** |  |
|  |  | (0.004) | (0.004) | (0.003) | (0.005) |  |
| 6.refmonth |  | (omitted) | | | | |
|  |  |  |  |  |  |  |
| 7.refmonth |  | -0.00140 | 0.00282 | -0.00291 | -0.00438 |  |
|  |  | (0.004) | (0.004) | (0.004) | (0.006) |  |
| 8.refmonth |  | -0.000274 | 0.00198 | -0.00225 | -0.00607 |  |
|  |  | (0.004) | (0.004) | (0.004) | (0.006) |  |
| 9.refmonth |  | -0.0112** | -0.00708* | -0.0108** | -0.00692 |  |
|  |  | (0.005) | (0.004) | (0.004) | (0.005) |  |
| 10.refmonth |  | -0.0122** | -0.00906** | -0.00832** | -0.00927* |  |
|  |  | (0.005) | (0.004) | (0.004) | (0.005) |  |
| 11.refmonth |  | -0.00623 | -0.00936** | -0.00598 | -0.00966* |  |
|  |  | (0.004) | (0.004) | (0.004) | (0.006) |  |
| 12.refmonth |  | -0.0128** | -0.0105** | -0.0186*** | -0.0126*** |  |
|  |  | (0.005) | (0.004) | (0.005) | (0.005) |  |
| 13.refmonth |  | -0.0157*** | -0.0134*** | -0.00759* | -0.0120** |  |
|  |  | (0.005) | (0.005) | (0.004) | (0.005) |  |
| _cons |  | 4.384*** | 4.394*** | 4.397*** | 4.395*** |  |
|  |  | (0.003) | (0.004) | (0.003) | (0.004) |  |
| N |  | 183603 | 325692 | 324892 | 240936 |  |
| Significance levels: * 10 percent, ** 5 percent, *** 1 percent. Standard errors (in parentheses) are clustered by county. Only estimated coefficients (and their standard errors) for interaction terms are used for the event-study plots. | | | | | | |

Results for Fig 2: Berry Index by classification of race/ethnicity

|  |  | Dependent variable: Natural log of Berry Index | | | | | |
| --- | --- | --- | --- | --- | --- | --- | --- |
| Independent variable | Relative month | (1) | (2) | (3) | (4) | (5) |  |
|  |  | Hispanic | White | Black | Asian | Others |  |
| 1.pandemicyear#1.refmonth | -6 | -0.0145 | -0.00854*** | -0.0103 | -0.0295 | 0.0183 |  |
|  |  | (0.012) | (0.003) | (0.015) | (0.024) | (0.018) |  |
| 1.pandemicyear#2.refmonth | -5 | -0.0159 | -0.00319 | -0.0100 | -0.0401** | 0.0283 |  |
|  |  | (0.014) | (0.003) | (0.011) | (0.016) | (0.024) |  |
| 1.pandemicyear#3.refmonth | -4 | -0.00770 | -0.00285 | -0.0116 | -0.00309 | 0.0136 |  |
|  |  | (0.013) | (0.003) | (0.012) | (0.014) | (0.018) |  |
| 1.pandemicyear#4.refmonth | -3 | 0.00619 | -0.00489 | -0.0192 | 0.00120 | 0.0224 |  |
|  |  | (0.013) | (0.003) | (0.013) | (0.015) | (0.019) |  |
| 1.pandemicyear#5.refmonth | -2 | -0.0205* | -0.00222 | -0.0197 | -0.000269 | 0.00204 |  |
|  |  | (0.012) | (0.003) | (0.013) | (0.020) | (0.032) |  |
| 1.pandemicyear#6.refmonth | -1 | (omitted) | | | | |  |
|  |  |  |  |  |  |  |  |
| 1.pandemicyear#7.refmonth | 0 | 0.0311** | 0.0227*** | 0.0354** | 0.0370** | 0.0461** |  |
|  |  | (0.016) | (0.003) | (0.014) | (0.017) | (0.018) |  |
| 1.pandemicyear#8.refmonth | 1 | 0.0295** | 0.0145*** | -0.00614 | 0.0192 | 0.0224 |  |
|  |  | (0.015) | (0.004) | (0.014) | (0.023) | (0.025) |  |
| 1.pandemicyear#9.refmonth | 2 | 0.00880 | 0.0220*** | 0.0216 | 0.0229 | 0.0570** |  |
|  |  | (0.014) | (0.004) | (0.013) | (0.017) | (0.024) |  |
| 1.pandemicyear#10.refmonth | 3 | 0.0288** | 0.00745** | 0.00943 | 0.0273 | 0.0505** |  |
|  |  | (0.014) | (0.004) | (0.013) | (0.017) | (0.025) |  |
| 1.pandemicyear#11.refmonth | 4 | 0.0137 | 0.0120*** | 0.00743 | 0.0126 | 0.0403** |  |
|  |  | (0.014) | (0.003) | (0.017) | (0.025) | (0.019) |  |
| 1.pandemicyear#12.refmonth | 5 | -0.00310 | 0.0141*** | 0.00201 | 0.0451** | 0.0150 |  |
|  |  | (0.016) | (0.004) | (0.015) | (0.021) | (0.026) |  |
| 1.pandemicyear#13.refmonth | 6 | 0.0101 | 0.0125*** | 0.0115 | 0.0313 | 0.0444** |  |
|  |  | (0.014) | (0.004) | (0.013) | (0.019) | (0.019) |  |
| 1.pandemicyear |  | -0.00184 | -0.00456* | 0.00888 | 0.00282 | -0.0218 |  |
|  |  | (0.008) | (0.002) | (0.011) | (0.014) | (0.017) |  |
| 1.refmonth |  | 0.000666 | 0.000274 | 0.00325 | 0.00541 | -0.00620 |  |
|  |  | (0.007) | (0.002) | (0.013) | (0.020) | (0.007) |  |
| 2.refmonth |  | -0.00121 | 0.00306 | 0.0201** | 0.0147 | -0.0182 |  |
|  |  | (0.009) | (0.002) | (0.009) | (0.013) | (0.017) |  |
| 3.refmonth |  | 0.0131 | 0.0103*** | 0.0272*** | 0.0155 | 0.00536 |  |
|  |  | (0.010) | (0.002) | (0.010) | (0.013) | (0.005) |  |
| 4.refmonth |  | -0.00735 | 0.00743*** | 0.0206** | 0.000333 | -0.0124 |  |
|  |  | (0.013) | (0.002) | (0.010) | (0.011) | (0.008) |  |
| 5.refmonth |  | 0.0147* | 0.00660*** | 0.0259*** | -0.00108 | -0.0131 |  |
|  |  | (0.008) | (0.002) | (0.010) | (0.016) | (0.017) |  |
| 6.refmonth |  | (omitted) | | | | |  |
|  |  |  |  |  |  |  |  |
| 7.refmonth |  | -0.00755 | -0.00105 | 0.000289 | 0.00283 | -0.00371 |  |
|  |  | (0.011) | (0.002) | (0.012) | (0.015) | (0.005) |  |
| 8.refmonth |  | -0.00954 | -0.00301 | 0.0193** | -0.0136 | -0.00543 |  |
|  |  | (0.011) | (0.002) | (0.009) | (0.016) | (0.006) |  |
| 9.refmonth |  | -0.00506 | -0.0115*** | 0.00688 | 0.00337 | -0.0212 |  |
|  |  | (0.009) | (0.002) | (0.009) | (0.013) | (0.017) |  |
| 10.refmonth |  | -0.0178* | -0.00948*** | 0.00160 | -0.0140 | -0.0270* |  |
|  |  | (0.010) | (0.002) | (0.010) | (0.013) | (0.015) |  |
| 11.refmonth |  | -0.0133 | -0.00749*** | -0.00265 | -0.0161 | -0.0169** |  |
|  |  | (0.010) | (0.002) | (0.011) | (0.017) | (0.007) |  |
| 12.refmonth |  | -0.00366 | -0.0157*** | 0.00185 | -0.0334* | -0.0202*** |  |
|  |  | (0.008) | (0.003) | (0.010) | (0.020) | (0.006) |  |
| 13.refmonth |  | -0.0119 | -0.0134*** | 0.00372 | -0.0143 | -0.0224*** |  |
|  |  | (0.011) | (0.002) | (0.011) | (0.017) | (0.007) |  |
| _cons |  | 4.381*** | 4.401*** | 4.358*** | 4.358*** | 4.400*** |  |
|  |  | (0.008) | (0.002) | (0.009) | (0.014) | (0.006) |  |
| N |  | 67598 | 829960 | 113534 | 37764 | 26267 |  |
| Significance levels: * 10 percent, ** 5 percent, *** 1 percent. Standard errors (in parentheses) are clustered by county. Only estimated coefficients (and their standard errors) for interaction terms are used for the event-study plots. | | | | | | |  |

Results for Fig 2: Berry Index by number of household income sources

|  |  | Dependent variable: Natural log of Berry Index | | | | |
| --- | --- | --- | --- | --- | --- | --- |
| Independent variable | Relative month | (1) | (2) | (3) |  |  |
|  |  | No income | Single income | Dual income |  |  |
| 1.pandemicyear#1.refmonth | -6 | -0.00714 | -0.00656 | -0.0156** |  |  |
|  |  | (0.005) | (0.006) | (0.006) |  |  |
| 1.pandemicyear#2.refmonth | -5 | -0.00434 | -0.00212 | -0.0110* |  |  |
|  |  | (0.005) | (0.005) | (0.006) |  |  |
| 1.pandemicyear#3.refmonth | -4 | -0.00712 | -0.00160 | -0.00286 |  |  |
|  |  | (0.004) | (0.005) | (0.006) |  |  |
| 1.pandemicyear#4.refmonth | -3 | -0.0106** | -0.000000745 | -0.00543 |  |  |
|  |  | (0.004) | (0.006) | (0.006) |  |  |
| 1.pandemicyear#5.refmonth | -2 | -0.00399 | -0.00245 | -0.0102* |  |  |
|  |  | (0.005) | (0.005) | (0.006) |  |  |
| 1.pandemicyear#6.refmonth | -1 | (omitted) | | | | |
|  |  |  |  |  |  |  |
| 1.pandemicyear#7.refmonth | 0 | 0.0147*** | 0.0345*** | 0.0251*** |  |  |
|  |  | (0.005) | (0.006) | (0.006) |  |  |
| 1.pandemicyear#8.refmonth | 1 | -0.00767 | 0.0235*** | 0.0237*** |  |  |
|  |  | (0.007) | (0.005) | (0.006) |  |  |
| 1.pandemicyear#9.refmonth | 2 | 0.0186*** | 0.0281*** | 0.0169*** |  |  |
|  |  | (0.006) | (0.006) | (0.006) |  |  |
| 1.pandemicyear#10.refmonth | 3 | 0.00423 | 0.0194*** | 0.00536 |  |  |
|  |  | (0.006) | (0.006) | (0.007) |  |  |
| 1.pandemicyear#11.refmonth | 4 | 0.00704 | 0.0207*** | 0.00592 |  |  |
|  |  | (0.005) | (0.006) | (0.006) |  |  |
| 1.pandemicyear#12.refmonth | 5 | 0.00287 | 0.0231*** | 0.00917 |  |  |
|  |  | (0.005) | (0.006) | (0.007) |  |  |
| 1.pandemicyear#13.refmonth | 6 | 0.00797 | 0.0186*** | 0.0130** |  |  |
|  |  | (0.005) | (0.006) | (0.006) |  |  |
| 1.pandemicyear |  | -0.000772 | -0.00598 | -0.00158 |  |  |
|  |  | (0.004) | (0.004) | (0.005) |  |  |
| 1.refmonth |  | 0.000612 | -0.000899 | 0.00300 |  |  |
|  |  | (0.003) | (0.004) | (0.004) |  |  |
| 2.refmonth |  | 0.00555 | 0.00356 | 0.00467 |  |  |
|  |  | (0.004) | (0.004) | (0.004) |  |  |
| 3.refmonth |  | 0.0136*** | 0.0113*** | 0.0125*** |  |  |
|  |  | (0.003) | (0.004) | (0.004) |  |  |
| 4.refmonth |  | 0.0123*** | 0.00276 | 0.00784* |  |  |
|  |  | (0.003) | (0.004) | (0.004) |  |  |
| 5.refmonth |  | 0.00500 | 0.00903** | 0.0114*** |  |  |
|  |  | (0.003) | (0.004) | (0.003) |  |  |
| 6.refmonth |  | (omitted) | | | | |
|  |  |  |  |  |  |  |
| 7.refmonth |  | 0.000294 | -0.00236 | -0.00139 |  |  |
|  |  | (0.003) | (0.004) | (0.004) |  |  |
| 8.refmonth |  | 0.00200 | -0.00161 | -0.00539 |  |  |
|  |  | (0.003) | (0.004) | (0.005) |  |  |
| 9.refmonth |  | -0.0103** | -0.0106*** | -0.00468 |  |  |
|  |  | (0.004) | (0.004) | (0.004) |  |  |
| 10.refmonth |  | -0.00776** | -0.0113*** | -0.00852** |  |  |
|  |  | (0.003) | (0.004) | (0.004) |  |  |
| 11.refmonth |  | -0.00402 | -0.0115*** | -0.00693* |  |  |
|  |  | (0.004) | (0.004) | (0.004) |  |  |
| 12.refmonth |  | -0.00789** | -0.0174*** | -0.0154*** |  |  |
|  |  | (0.003) | (0.004) | (0.005) |  |  |
| 13.refmonth |  | -0.00855** | -0.0139*** | -0.0123*** |  |  |
|  |  | (0.004) | (0.004) | (0.004) |  |  |
| _cons |  | 4.393*** | 4.384*** | 4.407*** |  |  |
|  |  | (0.003) | (0.003) | (0.004) |  |  |
| N |  | 343247 | 439100 | 292776 | # |  |
| Significance levels: * 10 percent, ** 5 percent, *** 1 percent. Standard errors (in parentheses) are clustered by county. Only estimated coefficients (and their standard errors) for interaction terms are used for the event-study plots. | | | | | | |

Results for Fig 2: Berry Index by vehicle ownership

|  |  | Dependent variable: Natural log of Berry Index | | | |  |
| --- | --- | --- | --- | --- | --- | --- |
| Independent variable | Relative month |  | (1) | (2) |  |  |
|  |  |  | Vehicle owner | Without vehicle |  |  |
| 1.pandemicyear#1.refmonth | -6 |  | -0.00798 | -0.00988** |  |  |
|  |  |  | (0.005) | (0.004) |  |  |
| 1.pandemicyear#2.refmonth | -5 |  | 0.00598 | -0.0114*** |  |  |
|  |  |  | (0.005) | (0.004) |  |  |
| 1.pandemicyear#3.refmonth | -4 |  | 0.00348 | -0.00761* |  |  |
|  |  |  | (0.004) | (0.004) |  |  |
| 1.pandemicyear#4.refmonth | -3 |  | 0.00789* | -0.0118*** |  |  |
|  |  |  | (0.005) | (0.004) |  |  |
| 1.pandemicyear#5.refmonth | -2 |  | 0.000520 | -0.00809** |  |  |
|  |  |  | (0.005) | (0.004) |  |  |
| 1.pandemicyear#6.refmonth | -1 |  | (omitted) | |  |  |
|  |  |  |  |  |  |  |
| 1.pandemicyear#7.refmonth | 0 |  | 0.0384*** | 0.0187*** |  |  |
|  |  |  | (0.004) | (0.004) |  |  |
| 1.pandemicyear#8.refmonth | 1 |  | 0.0371*** | 0.000788 |  |  |
|  |  |  | (0.005) | (0.005) |  |  |
| 1.pandemicyear#9.refmonth | 2 |  | 0.0337*** | 0.0157*** |  |  |
|  |  |  | (0.005) | (0.005) |  |  |
| 1.pandemicyear#10.refmonth | 3 |  | 0.0205*** | 0.00541 |  |  |
|  |  |  | (0.005) | (0.004) |  |  |
| 1.pandemicyear#11.refmonth | 4 |  | 0.0210*** | 0.00761* |  |  |
|  |  |  | (0.005) | (0.004) |  |  |
| 1.pandemicyear#12.refmonth | 5 |  | 0.0168*** | 0.0107** |  |  |
|  |  |  | (0.005) | (0.005) |  |  |
| 1.pandemicyear#13.refmonth | 6 |  | 0.0218*** | 0.00931** |  |  |
|  |  |  | (0.005) | (0.004) |  |  |
| 1.pandemicyear |  |  | -0.0106*** | 0.000950 |  |  |
|  |  |  | (0.004) | (0.003) |  |  |
| 1.refmonth |  |  | 0.00157 | 0.000145 |  |  |
|  |  |  | (0.002) | (0.003) |  |  |
| 2.refmonth |  |  | 0.00137 | 0.00620** |  |  |
|  |  |  | (0.003) | (0.003) |  |  |
| 3.refmonth |  |  | 0.0123*** | 0.0124*** |  |  |
|  |  |  | (0.002) | (0.003) |  |  |
| 4.refmonth |  |  | 0.00723** | 0.00714** |  |  |
|  |  |  | (0.003) | (0.003) |  |  |
| 5.refmonth |  |  | 0.00672** | 0.00931*** |  |  |
|  |  |  | (0.003) | (0.003) |  |  |
| 6.refmonth |  |  | (omitted) | |  |  |
|  |  |  |  |  |  |  |
| 7.refmonth |  |  | -0.00531* | 0.000959 |  |  |
|  |  |  | (0.003) | (0.003) |  |  |
| 8.refmonth |  |  | -0.00646** | 0.00122 |  |  |
|  |  |  | (0.003) | (0.003) |  |  |
| 9.refmonth |  |  | -0.00872*** | -0.00897*** |  |  |
|  |  |  | (0.003) | (0.003) |  |  |
| 10.refmonth |  |  | -0.0114*** | -0.00837*** |  |  |
|  |  |  | (0.003) | (0.003) |  |  |
| 11.refmonth |  |  | -0.00997*** | -0.00674** |  |  |
|  |  |  | (0.003) | (0.003) |  |  |
| 12.refmonth |  |  | -0.0107*** | -0.0155*** |  |  |
|  |  |  | (0.003) | (0.004) |  |  |
| 13.refmonth |  |  | -0.0127*** | -0.0112*** |  |  |
|  |  |  | (0.003) | (0.003) |  |  |
| _cons |  |  | 4.404*** | 4.388*** |  |  |
|  |  |  | (0.002) | (0.003) |  |  |
| N |  |  | 379449 | 695674 |  |  |
| Significance levels: * 10 percent, ** 5 percent, *** 1 percent. Standard errors (in parentheses) are clustered by county. Only estimated coefficients (and their standard errors) for interaction terms are used for the event-study plots. | | | | | |  |
