# Supplementary material for: Pandemic-induced changes in household-level food diversity and diet quality in the U.S
Source: PLoS One. 2024 May 31;19(5):e0300839. doi: 10.1371/journal.pone.0300839 (PMC11142597; doi:10.1371/journal.pone.0300839)
Supplement: S2 Appendix — (DOCX) [file pone.0300839.s003.docx]

**S3 Appendix. Results for USDAScore regressions with heterogeneity.**

Results for Fig 3: USDAScore by Census region of residence

|  |  | Dependent variable: Natural log of USDAScore1 | | | | |
| --- | --- | --- | --- | --- | --- | --- |
| Independent variable | Relative month | (1) | (2) | (3) | (4) |  |
|  |  | Northeast | Midwest | South | West |  |
| 1.pandemicyear#1.refmonth | -6 | 0.00224 | -0.00147 | -0.00684 | 0.00102 |  |
|  |  | (0.008) | (0.005) | (0.005) | (0.006) |  |
| 1.pandemicyear#2.refmonth | -5 | -0.0121* | 0.00565 | -0.00894* | -0.00629 |  |
|  |  | (0.007) | (0.005) | (0.005) | (0.006) |  |
| 1.pandemicyear#3.refmonth | -4 | 0.00470 | 0.00816 | -0.00246 | 0.00559 |  |
|  |  | (0.008) | (0.005) | (0.005) | (0.006) |  |
| 1.pandemicyear#4.refmonth | -3 | -0.000351 | 0.0000176 | -0.0117** | -0.0195*** |  |
|  |  | (0.007) | (0.006) | (0.005) | (0.007) |  |
| 1.pandemicyear#5.refmonth | -2 | -0.00375 | 0.00279 | -0.00862* | -0.000585 |  |
|  |  | (0.008) | (0.005) | (0.005) | (0.006) |  |
| 1.pandemicyear#6.refmonth | -1 | (omitted) | | | | |
|  |  |  |  |  |  |  |
| 1.pandemicyear#7.refmonth | 0 | 0.0780*** | 0.0728*** | 0.0757*** | 0.0805*** |  |
|  |  | (0.007) | (0.006) | (0.005) | (0.006) |  |
| 1.pandemicyear#8.refmonth | 1 | 0.0396*** | 0.0646*** | 0.0763*** | 0.0694*** |  |
|  |  | (0.008) | (0.005) | (0.005) | (0.007) |  |
| 1.pandemicyear#9.refmonth | 2 | 0.0779*** | 0.0935*** | 0.0867*** | 0.0746*** |  |
|  |  | (0.008) | (0.006) | (0.005) | (0.008) |  |
| 1.pandemicyear#10.refmonth | 3 | 0.0625*** | 0.0612*** | 0.0495*** | 0.0513*** |  |
|  |  | (0.008) | (0.006) | (0.005) | (0.007) |  |
| 1.pandemicyear#11.refmonth | 4 | 0.0398*** | 0.0369*** | 0.0377*** | 0.0426*** |  |
|  |  | (0.008) | (0.005) | (0.005) | (0.007) |  |
| 1.pandemicyear#12.refmonth | 5 | 0.0357*** | 0.0438*** | 0.0472*** | 0.0441*** |  |
|  |  | (0.008) | (0.006) | (0.005) | (0.008) |  |
| 1.pandemicyear#13.refmonth | 6 | 0.0190** | 0.0288*** | 0.0308*** | 0.0428*** |  |
|  |  | (0.008) | (0.006) | (0.005) | (0.008) |  |
| 1.pandemicyear |  | -0.0162*** | -0.0206*** | -0.0117*** | -0.00936* |  |
|  |  | (0.006) | (0.004) | (0.004) | (0.005) |  |
| 1.refmonth |  | -0.0414*** | -0.0335*** | -0.00596* | -0.0193*** |  |
|  |  | (0.005) | (0.004) | (0.003) | (0.005) |  |
| 2.refmonth |  | -0.0177*** | -0.0225*** | 0.00380 | -0.00512 |  |
|  |  | (0.004) | (0.004) | (0.004) | (0.005) |  |
| 3.refmonth |  | -0.00284 | 0.00667* | 0.0179*** | 0.0123*** |  |
|  |  | (0.005) | (0.004) | (0.003) | (0.005) |  |
| 4.refmonth |  | -0.00918* | -0.00757** | 0.000812 | 0.00420 |  |
|  |  | (0.005) | (0.004) | (0.004) | (0.005) |  |
| 5.refmonth |  | 0.0192*** | 0.0120*** | 0.0225*** | 0.00905** |  |
|  |  | (0.005) | (0.004) | (0.004) | (0.004) |  |
| 6.refmonth |  | (omitted) | | | | |
|  |  |  |  |  |  |  |
| 7.refmonth |  | -0.00622 | -0.00687* | -0.00170 | -0.00162 |  |
|  |  | (0.005) | (0.004) | (0.004) | (0.004) |  |
| 8.refmonth |  | -0.00940** | -0.0132*** | -0.00578 | -0.00865* |  |
|  |  | (0.005) | (0.004) | (0.004) | (0.005) |  |
| 9.refmonth |  | -0.0218*** | -0.0376*** | -0.0221*** | -0.0119** |  |
|  |  | (0.006) | (0.004) | (0.004) | (0.005) |  |
| 10.refmonth |  | -0.0291*** | -0.0324*** | -0.0126*** | -0.00842* |  |
|  |  | (0.006) | (0.004) | (0.004) | (0.005) |  |
| 11.refmonth |  | -0.00923* | -0.0238*** | -0.00582 | -0.00322 |  |
|  |  | (0.005) | (0.004) | (0.004) | (0.006) |  |
| 12.refmonth |  | -0.0421*** | -0.0507*** | -0.0270*** | -0.0231*** |  |
|  |  | (0.006) | (0.004) | (0.004) | (0.006) |  |
| 13.refmonth |  | -0.0498*** | -0.0522*** | -0.0206*** | -0.0319*** |  |
|  |  | (0.005) | (0.004) | (0.004) | (0.006) |  |
| _cons |  | 1.799*** | 1.765*** | 1.759*** | 1.768*** |  |
|  |  | (0.008) | (0.005) | (0.005) | (0.010) |  |
| N |  | 184810 | 274772 | 401403 | 205953 |  |
| Significance levels: * 10 percent, ** 5 percent, *** 1 percent. Standard errors (in parentheses) are clustered by county. Only estimated coefficients (and their standard errors) for interaction terms are used for the event-study plots. | | | | | | |

Results for Fig 3: USDAScore by age of children

|  |  | Dependent variable: Natural log of USDAScore1 | | | | |
| --- | --- | --- | --- | --- | --- | --- |
| Independent variable | Relative month | (1) | (2) | (3) | (4) |  |
|  |  | No children under 18 years old | Any young children | School-age children | Only middle-school or high-school children |  |
| 1.pandemicyear#1.refmonth | -6 | 0.000198 | -0.00852 | -0.0177 | -0.0144 |  |
|  |  | (0.003) | (0.016) | (0.011) | (0.014) |  |
| 1.pandemicyear#2.refmonth | -5 | -0.00458 | -0.0307* | -0.00482 | 0.00117 |  |
|  |  | (0.003) | (0.016) | (0.012) | (0.012) |  |
| 1.pandemicyear#3.refmonth | -4 | 0.00403 | -0.00665 | 0.00243 | -0.00380 |  |
|  |  | (0.003) | (0.016) | (0.012) | (0.012) |  |
| 1.pandemicyear#4.refmonth | -3 | -0.00688** | 0.00136 | -0.0150 | -0.0247** |  |
|  |  | (0.003) | (0.016) | (0.011) | (0.013) |  |
| 1.pandemicyear#5.refmonth | -2 | -0.00418 | -0.00460 | 0.00712 | -0.00278 |  |
|  |  | (0.003) | (0.014) | (0.010) | (0.012) |  |
| 1.pandemicyear#6.refmonth | -1 | (omitted) | | | | |
|  |  |  |  |  |  |  |
| 1.pandemicyear#7.refmonth | 0 | 0.0759*** | 0.0359** | 0.0837*** | 0.100*** |  |
|  |  | (0.003) | (0.016) | (0.012) | (0.012) |  |
| 1.pandemicyear#8.refmonth | 1 | 0.0648*** | 0.0332* | 0.0734*** | 0.0888*** |  |
|  |  | (0.003) | (0.018) | (0.012) | (0.013) |  |
| 1.pandemicyear#9.refmonth | 2 | 0.0874*** | 0.0370** | 0.0819*** | 0.0799*** |  |
|  |  | (0.004) | (0.017) | (0.012) | (0.014) |  |
| 1.pandemicyear#10.refmonth | 3 | 0.0561*** | 0.0243 | 0.0575*** | 0.0592*** |  |
|  |  | (0.003) | (0.018) | (0.012) | (0.014) |  |
| 1.pandemicyear#11.refmonth | 4 | 0.0379*** | 0.0460** | 0.0490*** | 0.0345** |  |
|  |  | (0.003) | (0.018) | (0.013) | (0.014) |  |
| 1.pandemicyear#12.refmonth | 5 | 0.0446*** | 0.0261 | 0.0440*** | 0.0438*** |  |
|  |  | (0.003) | (0.018) | (0.013) | (0.014) |  |
| 1.pandemicyear#13.refmonth | 6 | 0.0338*** | -0.00881 | 0.0168 | 0.0272** |  |
|  |  | (0.004) | (0.019) | (0.012) | (0.014) |  |
| 1.pandemicyear |  | -0.0158*** | 0.000702 | -0.0149* | -0.00278 |  |
|  |  | (0.002) | (0.012) | (0.008) | (0.009) |  |
| 1.refmonth |  | -0.0228*** | -0.0369*** | -0.0110 | -0.0108 |  |
|  |  | (0.002) | (0.011) | (0.008) | (0.009) |  |
| 2.refmonth |  | -0.00796*** | -0.0140 | -0.00706 | -0.0116 |  |
|  |  | (0.002) | (0.011) | (0.008) | (0.009) |  |
| 3.refmonth |  | 0.0117*** | -0.0141 | 0.00449 | 0.0159* |  |
|  |  | (0.002) | (0.011) | (0.008) | (0.009) |  |
| 4.refmonth |  | 0.0000731 | -0.0329*** | -0.0170** | 0.00160 |  |
|  |  | (0.002) | (0.012) | (0.008) | (0.009) |  |
| 5.refmonth |  | 0.0159*** | 0.0146 | 0.0245*** | 0.0184** |  |
|  |  | (0.002) | (0.011) | (0.008) | (0.008) |  |
| 6.refmonth |  | (omitted) | | | | |
|  |  |  |  |  |  |  |
| 7.refmonth |  | -0.00231 | -0.00404 | -0.0166** | -0.00817 |  |
|  |  | (0.002) | (0.011) | (0.008) | (0.009) |  |
| 8.refmonth |  | -0.00676*** | -0.0211* | -0.0173** | -0.0193** |  |
|  |  | (0.002) | (0.011) | (0.008) | (0.009) |  |
| 9.refmonth |  | -0.0243*** | -0.0245** | -0.0286*** | -0.0139 |  |
|  |  | (0.002) | (0.011) | (0.009) | (0.009) |  |
| 10.refmonth |  | -0.0173*** | -0.0387*** | -0.0310*** | -0.0267*** |  |
|  |  | (0.002) | (0.011) | (0.008) | (0.010) |  |
| 11.refmonth |  | -0.00545** | -0.0584*** | -0.0393*** | -0.0134 |  |
|  |  | (0.003) | (0.013) | (0.009) | (0.010) |  |
| 12.refmonth |  | -0.0319*** | -0.0642*** | -0.0491*** | -0.0399*** |  |
|  |  | (0.003) | (0.012) | (0.009) | (0.009) |  |
| 13.refmonth |  | -0.0351*** | -0.0527*** | -0.0424*** | -0.0285*** |  |
|  |  | (0.003) | (0.012) | (0.009) | (0.009) |  |
| _cons |  | 1.749*** | 1.901*** | 1.852*** | 1.861*** |  |
|  |  | (0.003) | (0.012) | (0.009) | (0.009) |  |
| N |  | 883384 | 41733 | 78562 | 63259 |  |
| Significance levels: * 10 percent, ** 5 percent, *** 1 percent. Standard errors (in parentheses) are clustered by county. Only estimated coefficients (and their standard errors) for interaction terms are used for the event-study plots. | | | | | | |

Results for Fig 3: USDAScore by household income level

|  |  | Dependent variable: Natural log of USDAScore1 | | | | |
| --- | --- | --- | --- | --- | --- | --- |
| Independent variable | Relative month | (1) | (2) | (3) | (4) |  |
|  |  | Low income | Low-Medium income | Medium-High income | High income |  |
| 1.pandemicyear#1.refmonth | -6 | -0.00135 | 0.00737 | -0.00579 | -0.0121* |  |
|  |  | (0.007) | (0.005) | (0.006) | (0.006) |  |
| 1.pandemicyear#2.refmonth | -5 | -0.00484 | -0.0109** | 0.00254 | -0.00850 |  |
|  |  | (0.007) | (0.005) | (0.005) | (0.007) |  |
| 1.pandemicyear#3.refmonth | -4 | 0.00259 | 0.00402 | 0.00592 | -0.00180 |  |
|  |  | (0.007) | (0.005) | (0.005) | (0.006) |  |
| 1.pandemicyear#4.refmonth | -3 | -0.0139* | -0.00960* | -0.00571 | -0.00589 |  |
|  |  | (0.007) | (0.005) | (0.005) | (0.006) |  |
| 1.pandemicyear#5.refmonth | -2 | 0.000668 | -0.00281 | -0.00527 | -0.00422 |  |
|  |  | (0.007) | (0.005) | (0.005) | (0.006) |  |
| 1.pandemicyear#6.refmonth | -1 | (omitted) | | | | |
|  |  |  |  |  |  |  |
| 1.pandemicyear#7.refmonth | 0 | 0.0565*** | 0.0656*** | 0.0894*** | 0.0879*** |  |
|  |  | (0.007) | (0.005) | (0.005) | (0.007) |  |
| 1.pandemicyear#8.refmonth | 1 | 0.0524*** | 0.0601*** | 0.0706*** | 0.0767*** |  |
|  |  | (0.008) | (0.006) | (0.006) | (0.007) |  |
| 1.pandemicyear#9.refmonth | 2 | 0.0680*** | 0.0750*** | 0.0914*** | 0.101*** |  |
|  |  | (0.008) | (0.005) | (0.006) | (0.007) |  |
| 1.pandemicyear#10.refmonth | 3 | 0.0382*** | 0.0485*** | 0.0649*** | 0.0634*** |  |
|  |  | (0.008) | (0.006) | (0.006) | (0.007) |  |
| 1.pandemicyear#11.refmonth | 4 | 0.0224*** | 0.0377*** | 0.0389*** | 0.0524*** |  |
|  |  | (0.007) | (0.005) | (0.006) | (0.007) |  |
| 1.pandemicyear#12.refmonth | 5 | 0.0232*** | 0.0360*** | 0.0508*** | 0.0600*** |  |
|  |  | (0.008) | (0.005) | (0.006) | (0.008) |  |
| 1.pandemicyear#13.refmonth | 6 | 0.0238*** | 0.0250*** | 0.0361*** | 0.0355*** |  |
|  |  | (0.008) | (0.005) | (0.006) | (0.008) |  |
| 1.pandemicyear |  | -0.0148*** | -0.0160*** | -0.0161*** | -0.00912* |  |
|  |  | (0.005) | (0.004) | (0.004) | (0.005) |  |
| 1.refmonth |  | -0.0108** | -0.0274*** | -0.0233*** | -0.0201*** |  |
|  |  | (0.005) | (0.004) | (0.004) | (0.005) |  |
| 2.refmonth |  | -0.000299 | -0.00391 | -0.0145*** | -0.0121*** |  |
|  |  | (0.005) | (0.004) | (0.004) | (0.005) |  |
| 3.refmonth |  | 0.0133** | 0.0118*** | 0.00504 | 0.0135*** |  |
|  |  | (0.005) | (0.004) | (0.004) | (0.005) |  |
| 4.refmonth |  | 0.000148 | -0.000211 | -0.00243 | -0.00696 |  |
|  |  | (0.005) | (0.004) | (0.004) | (0.005) |  |
| 5.refmonth |  | 0.00874* | 0.0145*** | 0.0162*** | 0.0260*** |  |
|  |  | (0.005) | (0.004) | (0.004) | (0.005) |  |
| 6.refmonth |  | (omitted) | | | | |
|  |  |  |  |  |  |  |
| 7.refmonth |  | -0.00144 | 0.000179 | -0.0110*** | -0.00100 |  |
|  |  | (0.005) | (0.004) | (0.004) | (0.005) |  |
| 8.refmonth |  | -0.00283 | -0.00846** | -0.0116*** | -0.00992** |  |
|  |  | (0.005) | (0.004) | (0.004) | (0.005) |  |
| 9.refmonth |  | -0.0129** | -0.0205*** | -0.0266*** | -0.0335*** |  |
|  |  | (0.005) | (0.004) | (0.004) | (0.005) |  |
| 10.refmonth |  | -0.0117** | -0.0200*** | -0.0217*** | -0.0225*** |  |
|  |  | (0.006) | (0.004) | (0.004) | (0.005) |  |
| 11.refmonth |  | 0.00176 | -0.0119*** | -0.0126*** | -0.0151*** |  |
|  |  | (0.005) | (0.004) | (0.004) | (0.005) |  |
| 12.refmonth |  | -0.0230*** | -0.0330*** | -0.0411*** | -0.0380*** |  |
|  |  | (0.006) | (0.004) | (0.004) | (0.005) |  |
| 13.refmonth |  | -0.0228*** | -0.0367*** | -0.0391*** | -0.0407*** |  |
|  |  | (0.005) | (0.004) | (0.004) | (0.005) |  |
| _cons |  | 1.656*** | 1.747*** | 1.804*** | 1.837*** |  |
|  |  | (0.006) | (0.005) | (0.005) | (0.006) |  |
| N |  | 181855 | 323160 | 322808 | 239115 |  |
| Significance levels: * 10 percent, ** 5 percent, *** 1 percent. Standard errors (in parentheses) are clustered by county. Only estimated coefficients (and their standard errors) for interaction terms are used for the event-study plots. | | | | | | |

Results for Fig 3: USDAScore by classification of race/ethnicity

|  |  | Dependent variable: Natural log of USDAScore1 | | | | | |
| --- | --- | --- | --- | --- | --- | --- | --- |
| Independent variable | Relative month | (1) | (2) | (3) | (4) | (5) |  |
|  |  | Hispanic | White | Black | Asian | Others |  |
| 1.pandemicyear#1.refmonth | -6 | 0.0237* | -0.00180 | -0.0111 | -0.0300* | -0.0111 |  |
|  |  | (0.013) | (0.003) | (0.011) | (0.017) | (0.022) |  |
| 1.pandemicyear#2.refmonth | -5 | 0.00240 | -0.00605* | 0.00965 | -0.0233 | -0.0383* |  |
|  |  | (0.012) | (0.003) | (0.010) | (0.018) | (0.022) |  |
| 1.pandemicyear#3.refmonth | -4 | 0.00180 | 0.00291 | 0.00883 | -0.00160 | -0.00839 |  |
|  |  | (0.013) | (0.003) | (0.011) | (0.020) | (0.019) |  |
| 1.pandemicyear#4.refmonth | -3 | -0.00569 | -0.00838*** | -0.00751 | 0.000379 | -0.0254 |  |
|  |  | (0.012) | (0.003) | (0.011) | (0.018) | (0.021) |  |
| 1.pandemicyear#5.refmonth | -2 | 0.00937 | -0.00554* | 0.00495 | 0.00705 | -0.0165 |  |
|  |  | (0.012) | (0.003) | (0.010) | (0.020) | (0.022) |  |
| 1.pandemicyear#6.refmonth | -1 | (omitted) | | | | |  |
|  |  |  |  |  |  |  |  |
| 1.pandemicyear#7.refmonth | 0 | 0.0933*** | 0.0700*** | 0.103*** | 0.113*** | 0.0628*** |  |
|  |  | (0.013) | (0.003) | (0.010) | (0.020) | (0.021) |  |
| 1.pandemicyear#8.refmonth | 1 | 0.105*** | 0.0560*** | 0.101*** | 0.108*** | 0.0558** |  |
|  |  | (0.013) | (0.003) | (0.012) | (0.020) | (0.022) |  |
| 1.pandemicyear#9.refmonth | 2 | 0.103*** | 0.0739*** | 0.137*** | 0.147*** | 0.0611*** |  |
|  |  | (0.014) | (0.003) | (0.012) | (0.019) | (0.022) |  |
| 1.pandemicyear#10.refmonth | 3 | 0.0826*** | 0.0451*** | 0.0879*** | 0.141*** | 0.0368* |  |
|  |  | (0.015) | (0.003) | (0.011) | (0.021) | (0.020) |  |
| 1.pandemicyear#11.refmonth | 4 | 0.0556*** | 0.0301*** | 0.0713*** | 0.102*** | 0.0384* |  |
|  |  | (0.015) | (0.003) | (0.010) | (0.020) | (0.021) |  |
| 1.pandemicyear#12.refmonth | 5 | 0.0666*** | 0.0338*** | 0.0896*** | 0.0961*** | 0.0247 |  |
|  |  | (0.015) | (0.003) | (0.013) | (0.021) | (0.022) |  |
| 1.pandemicyear#13.refmonth | 6 | 0.0440*** | 0.0242*** | 0.0538*** | 0.0830*** | 0.0190 |  |
|  |  | (0.014) | (0.003) | (0.011) | (0.019) | (0.023) |  |
| 1.pandemicyear |  | -0.0243** | -0.0146*** | -0.0127 | -0.00907 | 0.00787 |  |
|  |  | (0.011) | (0.002) | (0.008) | (0.014) | (0.015) |  |
| 1.refmonth |  | -0.0234** | -0.0234*** | -0.0102 | -0.0248* | -0.0109 |  |
|  |  | (0.010) | (0.002) | (0.007) | (0.014) | (0.016) |  |
| 2.refmonth |  | -0.0147 | -0.00632** | -0.0167** | -0.0218 | -0.00190 |  |
|  |  | (0.010) | (0.002) | (0.007) | (0.013) | (0.015) |  |
| 3.refmonth |  | 0.00962 | 0.0105*** | 0.0152** | -0.00307 | 0.00491 |  |
|  |  | (0.009) | (0.002) | (0.007) | (0.014) | (0.013) |  |
| 4.refmonth |  | -0.0195** | 0.00139 | -0.00378 | -0.0436*** | -0.0139 |  |
|  |  | (0.009) | (0.002) | (0.008) | (0.014) | (0.016) |  |
| 5.refmonth |  | 0.0188** | 0.0165*** | 0.0222*** | 0.000887 | 0.0169 |  |
|  |  | (0.009) | (0.002) | (0.007) | (0.014) | (0.016) |  |
| 6.refmonth |  | (omitted) | | | | |  |
|  |  |  |  |  |  |  |  |
| 7.refmonth |  | 0.00194 | -0.00425* | -0.00957 | 0.00421 | 0.00995 |  |
|  |  | (0.009) | (0.002) | (0.007) | (0.015) | (0.015) |  |
| 8.refmonth |  | -0.0192** | -0.00854*** | -0.00198 | -0.0237* | -0.000601 |  |
|  |  | (0.010) | (0.002) | (0.008) | (0.014) | (0.016) |  |
| 9.refmonth |  | -0.0260*** | -0.0235*** | -0.0304*** | -0.0249* | -0.00683 |  |
|  |  | (0.010) | (0.003) | (0.008) | (0.013) | (0.016) |  |
| 10.refmonth |  | -0.0341*** | -0.0178*** | -0.0225*** | -0.0413*** | -0.00207 |  |
|  |  | (0.011) | (0.002) | (0.008) | (0.015) | (0.016) |  |
| 11.refmonth |  | -0.0208* | -0.00859*** | -0.0128* | -0.0242* | -0.0158 |  |
|  |  | (0.012) | (0.003) | (0.008) | (0.014) | (0.015) |  |
| 12.refmonth |  | -0.0411*** | -0.0329*** | -0.0406*** | -0.0565*** | -0.0282* |  |
|  |  | (0.012) | (0.003) | (0.008) | (0.014) | (0.015) |  |
| 13.refmonth |  | -0.0410*** | -0.0364*** | -0.0257*** | -0.0505*** | -0.0314* |  |
|  |  | (0.011) | (0.002) | (0.007) | (0.015) | (0.016) |  |
| _cons |  | 1.803*** | 1.772*** | 1.722*** | 1.793*** | 1.758*** |  |
|  |  | (0.013) | (0.003) | (0.008) | (0.019) | (0.018) |  |
| N |  | 67079 | 823708 | 112689 | 37483 | 25979 |  |
| Significance levels: * 10 percent, ** 5 percent, *** 1 percent. Standard errors (in parentheses) are clustered by county. Only estimated coefficients (and their standard errors) for interaction terms are used for the event-study plots. | | | | | | |  |

Results for Fig 3: USDAScore by number of household income sources

|  |  | Dependent variable: Natural log of USDAScore1 | | | | |
| --- | --- | --- | --- | --- | --- | --- |
| Independent variable | Relative month | (1) | (2) | (3) |  |  |
|  |  | No income | Single income | Dual income |  |  |
| 1.pandemicyear#1.refmonth | -6 | 0.00507 | 0.000172 | -0.0149** |  |  |
|  |  | (0.005) | (0.005) | (0.006) |  |  |
| 1.pandemicyear#2.refmonth | -5 | -0.00425 | -0.00531 | -0.00645 |  |  |
|  |  | (0.005) | (0.005) | (0.006) |  |  |
| 1.pandemicyear#3.refmonth | -4 | 0.00524 | 0.00229 | 0.00167 |  |  |
|  |  | (0.005) | (0.005) | (0.006) |  |  |
| 1.pandemicyear#4.refmonth | -3 | -0.0142*** | -0.00505 | -0.00600 |  |  |
|  |  | (0.005) | (0.005) | (0.006) |  |  |
| 1.pandemicyear#5.refmonth | -2 | -0.00237 | -0.00112 | -0.00789 |  |  |
|  |  | (0.005) | (0.004) | (0.006) |  |  |
| 1.pandemicyear#6.refmonth | -1 | (omitted) | | | | |
|  |  |  |  |  |  |  |
| 1.pandemicyear#7.refmonth | 0 | 0.0555*** | 0.0905*** | 0.0790*** |  |  |
|  |  | (0.005) | (0.005) | (0.006) |  |  |
| 1.pandemicyear#8.refmonth | 1 | 0.0476*** | 0.0767*** | 0.0698*** |  |  |
|  |  | (0.006) | (0.005) | (0.006) |  |  |
| 1.pandemicyear#9.refmonth | 2 | 0.0743*** | 0.0912*** | 0.0865*** |  |  |
|  |  | (0.006) | (0.005) | (0.006) |  |  |
| 1.pandemicyear#10.refmonth | 3 | 0.0434*** | 0.0654*** | 0.0534*** |  |  |
|  |  | (0.006) | (0.005) | (0.006) |  |  |
| 1.pandemicyear#11.refmonth | 4 | 0.0347*** | 0.0455*** | 0.0335*** |  |  |
|  |  | (0.005) | (0.005) | (0.006) |  |  |
| 1.pandemicyear#12.refmonth | 5 | 0.0351*** | 0.0532*** | 0.0395*** |  |  |
|  |  | (0.006) | (0.005) | (0.007) |  |  |
| 1.pandemicyear#13.refmonth | 6 | 0.0289*** | 0.0357*** | 0.0245*** |  |  |
|  |  | (0.005) | (0.005) | (0.006) |  |  |
| 1.pandemicyear |  | -0.0157*** | -0.0161*** | -0.00995** |  |  |
|  |  | (0.004) | (0.003) | (0.004) |  |  |
| 1.refmonth |  | -0.0212*** | -0.0209*** | -0.0237*** |  |  |
|  |  | (0.004) | (0.003) | (0.004) |  |  |
| 2.refmonth |  | -0.00274 | -0.00768** | -0.0159*** |  |  |
|  |  | (0.004) | (0.003) | (0.004) |  |  |
| 3.refmonth |  | 0.0168*** | 0.00787** | 0.00651 |  |  |
|  |  | (0.004) | (0.003) | (0.004) |  |  |
| 4.refmonth |  | 0.00571 | -0.00771** | -0.00391 |  |  |
|  |  | (0.004) | (0.003) | (0.004) |  |  |
| 5.refmonth |  | 0.0126*** | 0.0167*** | 0.0214*** |  |  |
|  |  | (0.004) | (0.003) | (0.004) |  |  |
| 6.refmonth |  | (omitted) | | | | |
|  |  |  |  |  |  |  |
| 7.refmonth |  | -0.000118 | -0.00716** | -0.00285 |  |  |
|  |  | (0.004) | (0.003) | (0.004) |  |  |
| 8.refmonth |  | -0.00599* | -0.0123*** | -0.00684 |  |  |
|  |  | (0.004) | (0.003) | (0.004) |  |  |
| 9.refmonth |  | -0.0203*** | -0.0254*** | -0.0262*** |  |  |
|  |  | (0.004) | (0.004) | (0.004) |  |  |
| 10.refmonth |  | -0.0135*** | -0.0234*** | -0.0214*** |  |  |
|  |  | (0.004) | (0.004) | (0.005) |  |  |
| 11.refmonth |  | -0.00338 | -0.0127*** | -0.0156*** |  |  |
|  |  | (0.004) | (0.004) | (0.004) |  |  |
| 12.refmonth |  | -0.0272*** | -0.0363*** | -0.0417*** |  |  |
|  |  | (0.004) | (0.003) | (0.005) |  |  |
| 13.refmonth |  | -0.0279*** | -0.0370*** | -0.0436*** |  |  |
|  |  | (0.004) | (0.004) | (0.005) |  |  |
| _cons |  | 1.732*** | 1.737*** | 1.860*** |  |  |
|  |  | (0.005) | (0.004) | (0.005) |  |  |
| N |  | 340348 | 435932 | 290658 |  |  |
| Significance levels: * 10 percent, ** 5 percent, *** 1 percent. Standard errors (in parentheses) are clustered by county. Only estimated coefficients (and their standard errors) for interaction terms are used for the event-study plots. | | | | | | |

Results for Fig 3: USDAScore by vehicle ownership

|  |  | Dependent variable: Natural log of USDAScore1 | | | |  |
| --- | --- | --- | --- | --- | --- | --- |
| Independent variable | Relative month |  | (1) | (2) |  |  |
|  |  |  | Vehicle owner | Without vehicle |  |  |
| 1.pandemicyear#1.refmonth | -6 |  | -0.00298 | -0.00205 |  |  |
|  |  |  | (0.005) | (0.004) |  |  |
| 1.pandemicyear#2.refmonth | -5 |  | -0.00756 | -0.00396 |  |  |
|  |  |  | (0.005) | (0.004) |  |  |
| 1.pandemicyear#3.refmonth | -4 |  | 0.000521 | 0.00441 |  |  |
|  |  |  | (0.005) | (0.004) |  |  |
| 1.pandemicyear#4.refmonth | -3 |  | -0.00548 | -0.00970*** |  |  |
|  |  |  | (0.004) | (0.004) |  |  |
| 1.pandemicyear#5.refmonth | -2 |  | -0.00289 | -0.00353 |  |  |
|  |  |  | (0.005) | (0.004) |  |  |
| 1.pandemicyear#6.refmonth | -1 |  | (omitted) | |  |  |
|  |  |  |  |  |  |  |
| 1.pandemicyear#7.refmonth | 0 |  | 0.0871*** | 0.0704*** |  |  |
|  |  |  | (0.005) | (0.004) |  |  |
| 1.pandemicyear#8.refmonth | 1 |  | 0.0876*** | 0.0533*** |  |  |
|  |  |  | (0.005) | (0.004) |  |  |
| 1.pandemicyear#9.refmonth | 2 |  | 0.0951*** | 0.0788*** |  |  |
|  |  |  | (0.005) | (0.004) |  |  |
| 1.pandemicyear#10.refmonth | 3 |  | 0.0571*** | 0.0540*** |  |  |
|  |  |  | (0.005) | (0.004) |  |  |
| 1.pandemicyear#11.refmonth | 4 |  | 0.0432*** | 0.0364*** |  |  |
|  |  |  | (0.005) | (0.004) |  |  |
| 1.pandemicyear#12.refmonth | 5 |  | 0.0446*** | 0.0432*** |  |  |
|  |  |  | (0.005) | (0.004) |  |  |
| 1.pandemicyear#13.refmonth | 6 |  | 0.0314*** | 0.0300*** |  |  |
|  |  |  | (0.005) | (0.004) |  |  |
| 1.pandemicyear |  |  | -0.0162*** | -0.0133*** |  |  |
|  |  |  | (0.004) | (0.003) |  |  |
| 1.refmonth |  |  | -0.0237*** | -0.0207*** |  |  |
|  |  |  | (0.003) | (0.003) |  |  |
| 2.refmonth |  |  | -0.00645* | -0.00945*** |  |  |
|  |  |  | (0.003) | (0.003) |  |  |
| 3.refmonth |  |  | 0.0156*** | 0.00750*** |  |  |
|  |  |  | (0.003) | (0.003) |  |  |
| 4.refmonth |  |  | 0.00429 | -0.00611** |  |  |
|  |  |  | (0.003) | (0.003) |  |  |
| 5.refmonth |  |  | 0.0177*** | 0.0161*** |  |  |
|  |  |  | (0.003) | (0.003) |  |  |
| 6.refmonth |  |  | (omitted) | |  |  |
|  |  |  |  |  |  |  |
| 7.refmonth |  |  | -0.00740** | -0.00179 |  |  |
|  |  |  | (0.003) | (0.003) |  |  |
| 8.refmonth |  |  | -0.00857** | -0.00899*** |  |  |
|  |  |  | (0.003) | (0.003) |  |  |
| 9.refmonth |  |  | -0.0244*** | -0.0239*** |  |  |
|  |  |  | (0.004) | (0.003) |  |  |
| 10.refmonth |  |  | -0.0191*** | -0.0201*** |  |  |
|  |  |  | (0.004) | (0.003) |  |  |
| 11.refmonth |  |  | -0.0120*** | -0.00972*** |  |  |
|  |  |  | (0.003) | (0.003) |  |  |
| 12.refmonth |  |  | -0.0335*** | -0.0357*** |  |  |
|  |  |  | (0.003) | (0.003) |  |  |
| 13.refmonth |  |  | -0.0380*** | -0.0348*** |  |  |
|  |  |  | (0.004) | (0.003) |  |  |
| _cons |  |  | 1.785*** | 1.760*** |  |  |
|  |  |  | (0.004) | (0.004) |  |  |
| N |  |  | 377368 | 689570 |  |  |
| Significance levels: * 10 percent, ** 5 percent, *** 1 percent. Standard errors (in parentheses) are clustered by county. Only estimated coefficients (and their standard errors) for interaction terms are used for the event-study plots. | | | | | |  |
