# Supplementary material for: Pandemic-induced changes in household-level food diversity and diet quality in the U.S
Source: PLoS One. 2024 May 31;19(5):e0300839. doi: 10.1371/journal.pone.0300839 (PMC11142597; doi:10.1371/journal.pone.0300839)
Supplement: S3 Appendix — (PDF) [file pone.0300839.s004.pdf]

S4 Appendix. Definition of Household Demographic Variables and Summary Statistics

Demographic variable	*Values*	*Details*	*# households*	*% of households sample*
Census region of residence	**Northeast**	New England<br>Middle Atlantic	7,207	17.33
	**Midwest**	East North Central<br>West North Central	10,692	25.71
	**South**	South Atlantic<br>East South Central<br>West South Central	15,652	37.64
	**West**	Mountain<br>Pacific	8,028	19.31
Age of children<br><br>[Nielsen Panel categorical variable *age_and_presence_of_children*]	**No children under 18**	Households with no children.	34,413	82.77
	**Any young children**	Households with one or more child and all are younger than prinary school age	1,629	3.92
	**Mix of school-age children**	Households with more than child where at least one childe is younger than primary school age	3,070	7.38
	**Only middle-school children and older**	Household has one or more child but all are are middle school age or orlder.	2,467	5.93
Household income level<br><br>]	**Low**	$0 to $29,999	7,107	17.09
	**Low-Medium**	$30,000 to $59,999	12,587	30.27
	**Medium-High**	$60,000 to $99,999	12,561	30.21
	**High**	$100,000 and higher	9,324	22.42
Classification of race/ethnicity	**Others**	Non-hispanic Others	1,017	2.45
	**Hispanic**	Of hispanic origin, regardless of race	2,620	6.30
	**White**	Non-hispanic White	32,076	77.14
	**Black**	Non-hispanic Black	4,403	10.59
	**Asian**	Non-hispanic Asian	1,463	3.52
Number of income sources	**No income**	Households where no household head is employed	13,270	31.92
	**Single income**	Households with only one employed household head.	16,997	40.88
	**Dual income**	Households with two employed household head.	11,312	27.21
Vehicle ownership	**Without vehicle**	Households that never made purchases of items under "Automotive" category or made purchases in only 1 year out of 3 consecutive years (2018, 2019, 2020)	26,932	64.77
	**Vehicle owner**	Households that made purchases of items under "Automotive" category in at least 2 out of 3 consecutive years (2018, 2019, 2020)	14,647	35.23

Note: There are a total fo **41,579** households in the sample.
