# Supplementary material for: Pandemic-induced changes in household-level food diversity and diet quality in the U.S
Source: PLoS One. 2024 May 31;19(5):e0300839. doi: 10.1371/journal.pone.0300839 (PMC11142597; doi:10.1371/journal.pone.0300839)
Supplement: Attachment — Submitted filename: Response to Reviewers.pdf [file pone.0300839.s008.pdf]

Response to Reviewers (Manuscript PONE-D-23-26121)

We greatly appreciate the opportunity to revise our manuscript and thank the editor and three anonymous reviewers for their helpful and constructive comments and suggestions. We present our responses to this feedback in four sections: We start with a section that summarizes the main revisions and follow that with detailed responses to each of the three reviewers.

Summary of major revisions (as suggested by all three reviewers)

1. We have expanded the Abstract to include substantially more details
2. We included more details of the previous research most relevant to our study.
3. We provided more details about the data and the sample. For example, (i) we note the number of households in our study, (ii) we were clearer about the dates of the study period, (iii) we included a new table that presented the month-by-month averages of the *Berry Index* and the *USDAScore*, and we include more description of the demographic information of these households.
4. We corrected some notation in our estimated equation (1) and the equations that define the *Berry Index* and the *USDAScore*.
5. We included one additional set of caveats to our results that deal with concerns about stockpiling and food price inflation.

The following responses to individual reviewers, which we label as Reviewer 1, 2, and 3, follow next. In each case, we copy the reviewers concerns into this document and respond to them one by one.

Response to Reviewer 1

General concerns – abstract: *The abstract would benefit from providing more summary information about the study (specific suggestions are detailed below).*

> We are happy to expand the abstract and we have generally followed Reviewer 1's comments that appear below in the specific concerns section. More specifically, we (a) added a more details about the time period of the study (which drew on data from August 2018 through September 2020, but focused on the post pandemic period that generally ran from March through Augst 2020) to help readers put the event study in context, (b) added a detailed sentence about methods and data, (c) clarified that the percent changes were true percentages rather than percentage points, and (d) added a final sentence that explains some heterogeneous effects across household types.

General concerns – brief introduction: *The introduction is rather brief and could include more of the literature on dietary changes during the pandemic. Where individual studies are referred to from a secondary source (e.g. a systematic review), the original study should be cited/referenced.*

> Regarding this concern, we made several changes to the revised manuscript to add more details and content to the introduction. First, we added more details from the dietary quality studies that we originally cited. These new details include information about how dietary quality changes were measured or characterized. Second, we add citations for and brief discussion of two U.S.-based studies that we referred to in (in passing) the original manuscript but we did not cite. The new citations are [12 and 13] in the revised manuscript.
>
> On the other hand, we did not add additional discussion of more pandemic-related diet quality studies. As the reviewer probably knows, there are a great number of studies that investigate dietary behavior during the pandemic, and many of the results of these studies are mixed. We started drafting an addition paragraph on these additional studies as Reviewer 1 suggested but found that our narrative was losing focus. Thus, rather than include summaries of additional studies, we chose to focus on those that were most relevant to our study: namely, the British study that (in part) uses scanner data as we do, and studies focused on the U.S. or Canada.

General concerns – verb tense: *There was inconsistent use of past and present tense throughout the manuscript. Past tense throughout would be preferable.*

> Thank you for catching our inconsistencies. Unlike the review, however, we find it easiest and preferable to use present tense throughout. More specifically, we use the past tense only for the very first paragraph with describes what actually happened during the pandemic. We consistently use the present tense after that, when we discuss either our own study or other studies. Assuming we achieved consistency (and did not err), we hope the reviewer is okay with these choices.

General concerns – disposable income changes: *Discussion Lines 289 Some households (those where job security or household income was not affected) may have had more disposable income*

due to savings in costs for transportation, childcare and recreation. Would greater disposable income contribute to selection of more healthful food choices since these are sometimes more costly relative to foods high in energy and low in nutrients?

Reviewer 1 makes a good point regarding the notion that, for some households, higher disposable income may come along with less-binding time constraints. With this point in mind, we added extra text to the last paragraph of the Discussion section.

Specific concerns:

Abstract

Add time points (year/month) for pre-pandemic measures vs pandemic measures of diet quality. If this is not possible because of the varying timing across states, suggest adding text along the lines of what is written in the methods e.g. 'from 6 months before to 6 months after the event month'.. of school-closure in each state in response to the pandemic.

Done: We followed this suggestion and added some time points. For example, we stressed that the data span the August 2018 to September 2020 period, and the three months after he start of the school closure period generally correspond to the March-June 2020, depending on the specific household.

Provide an indication of sample size/number households from which data were obtained.

Done. We added a note that says 41,579 households comprise the final NielsenIQ sample after accounting for households that moved location or left the panel.

Lines 19 & 20 Clarify whether percentages are 'percent' or 'percentage points'.

Since the dependent variable in the estimated regressions are in log form, these percentages are true percent changes (and not an increase in percentage points). We added a minor change to emphasize this, but if the reviewer things of an even better way to make this clear, we would consider additional changes.

State what statistical analyses were performed.

Done. We added a sentence that specifically refers to the estimation of an event study.

Lines 307-309 of the conclusion would be relevant to include in the abstract.

Done. We have now included this text in the abstract.

Introduction

Line 26, for context it would be helpful to add when (year & month) the majority of states closed restaurants and cafeterias.

Done. We added the text "… starting in March 2020…"

Line 39-40 'the pandemic-induced closing of dine-in restaurants' presumably takeaway restaurants and food outlets remained open throughout? This could be stated to make it clear

that potentially 'unhealthy' or ready-to-eat foods were still available, assuming this was the case in most states.

While it is true that many takeaway restaurants and food outlets in the U.S. remained open during the pandemic, we are unable to find reliable statistics that describe the exact details of which restaurants remained open, which closed completely, and which remained open for take-out or delivery. However, we do cite a reliable study [1] that found that restaurant sales dropped by one-third during the pandemic. This new citation follows a citation of a study [6] that showed diet quality is higher for U.S. adults who eat away from home less frequently. We put these to findings together to draw our conclusion about behavioral changes during the pandemic.

More specifically, to avoid confusion over closure details for dine-in vs takeaway options, we have altered the relevant text. Now, we emphasize the decline in restaurant sales by citing [1] and note that this decline in restaurant sales plus the study about poorer diet quality for frequent away-from-home eaters [6] together create the logical possibility that the pandemic could positively affect diet quality. In other words, we have subtly shifted our language from the closure of restaurants to decline in restaurant sales.

Line 49 – it would be helpful to add how diet quality was measured in the study reference [9], and which diet quality measures were used for the four US studies.

Done. More specifically, we added new language stating that [9] uses the HEI,]10 and 11] use dietary recall of quantities in specific food categories, [new 12] specifically examines added sugars, and [new 13] uses survey participant agreement with the following statement, "Since COVID-19, I am eating more healthy foods".

Line 49- add the citations for all four of the US studies i.e. add the remaining two in addition to ref [10,11].

Done. We now have a new [12] and [13].

Line 59 could 'expenditure shares' be explained in simple terms on first use, e.g. 'share spent on a food category out of the total household purchase expenditure'?

Yes. Because this comment/question implies that the original text is unclear about this part of the definition, we added the following text: "…, where each share is the portion of category expenditure relative to total household purchase expenditure."

Line 58 to 79 – for articles on nutrition, these details on the construction of a dietary index may fit better in the methods section rather than the introduction.

We agree that construction of the *Berry index* and the *USDAScore* are better fits for the methods section. That section does, in fact, include even more details on their construction. However, since we very much want to mention these metrics in the instruction, we felt a need to define them in a brief but (hopefully) clear way. For that reason, we are choosing to keep the current text in in introductory section.

<u>*Materials and methods*</u>

Line 85 – 'data' is usually written in the plural, ie. 'these data allow the study' instead of 'this data'

Agreed. We made this fix plus a couple more that we found.

Line 145-146 -Is the share based on total household purchase expenditures, including non-food items, and how might this have changed during the pandemic (i.e. purchasing more non-food items from a single retail outlet to avoid multiple contacts/exposures)?

This is a good question. However, we exclude non-food expenditures, so our total expenditures calculations are really total food expenditures. We tried to make this clearer in our revised manuscript by changing "actual household purchase expenditures" to "actual household food-purchase expenditures".

Line 147: Has CNPP been defined in full previously? Same for the Table 1 heading.

We use the abbreviation CNPP in (original) line 147 and Table 1 because it was earlier defined as the Center for Nutrition Policy and Promotion on (original) lines 59-60. If the reviewer (or editor) feels readers need another reminder about the definition of CNPP, we will gladly add it.

Table 1: I wasn't clear where the 'Average actual monthly expenditure' share comes from in the column heading. For what year, or sample- is this the same 80,000 households over the full data period before dividing into pre-and post pandemic? Please ensure that Table 1 can be fully understood without needing additional information from the manuscript.

To improve the clarity of Table 1 and its accompanying text, we first added additional information to the title. We also added text that specifically notes the number of households in our sample and the time frame for the calculations.

Line 197- is there a word missing in 'each household's monthly score their score'?

Yes, there were one (or two!) words missing here. Thanks for catching it. To make this text as clear as possible, we now write, "where each household's monthly score is relative to their score in the previous year."

Line 217 and 230 Change 'white households' and 'Asian households' to 'Households classified as white ethnicity…etc

Done.

Line 240 – show the evidence that food purchasing behaviour of US household was dramatically affected. I couldn't see that this paper has shown dramatic changes in behaviour per se, but they may have changed food expenditure patterns. It would be incorrect to assume that this led directly to changes in food consumption patterns, since this has not been assessed.

To be honest, we are not completely clear what the reviewer is asking about or concerned about. It is clear that the reader is okay with us saying that we find pandemic induced changes in food expenditures. If so, then changes in food expenditures is also consistent with saying there was changes in food purchasing behavior, right? So, if we are following the logic correctly, the first part of the above concern is really not a concern.

It is also generally unwise to make claims about consumption patterns because our data are based on purchases not true consumption. Thus, we have tried hard to keep the focus on food purchases, food-group expenditures, food group expenditure shares, and the *Berry Index* and *USDAScore* calculations rather than consumption.

Lastly, it’s possible that the reviewer is concerned about any claim about long-term, lasting behavioral changes. However, don’t believe we have made any long-term claims.

If this section needs further clarification, we would be happy to oblige.

Figures 2 and 3: Is ‘race’ the appropriate term for figure headings (d)? Conventions vary in different global regions, ‘ethnicity’ may be an alternative term to use. The journal may give some guidance on this.

We will change “(d) Race” to “(d) Classification of Race/Ethnicity” but also seek guidance from the journal if necessary.

Response to Reviewer 2

Please refer to the introductory section of this report to read how appreciative we are for the reviewers' comments and to see a summary of our major revisions. The following section addresses Reviewer 2's comments one by one.

In the conclusion and discussion section, the proposed mechanism of decreased FAFH availability seems outside the scope of this study. As correctly noted, the present measures do not include any FAFH purchases and the study provides no evidence on the prevalence of restaurant closures or FAFH availability during these periods across states. Anecdotally, FAFH available for takeout and delivery appears to have been widely available during this period. Also, pre-prepared foods at grocery retailers may have similar health attributes as FAFH and could have been included in these grocery baskets. Again, I think this mechanism is somewhat of a stretch given the limits of the data presented in this article.

We think we understand Reviewer 2's concern here. The logic, as we understand it, is that many FAFH establishments still operated during the pandemic and that many supermarkets offer prepared meals that are similar in content to FAFH options. Thus, Reviewer 2 seems to be cautioning us against drawing any conclusions related to FAFH based on availability. Yet, we do not think Reviewer 2 is claiming that FAFH establishments were unaffected by the pandemic. Nor do we think Reviewer 2 is claiming that consumers had the full range of FAFH options during the pandemic.

We acknowledge that there is anecdotal evidence of at least some (maybe a lot) of FAFH availability during the pandemic via takeout and delivery options. Again, anecdotally, the availability of takeout/delivery from FAFH establishments seemed to partly depend on each state's definition of essential businesses and on whether or not there was a state decree on non-essential business closures. Thus, we believe (based on our own anecdotal observations) that at least some or even many restaurant options were, in fact, closed. We have looked for non-anecdotal evidence and documentation for FAFH availability but have failed to find reports that offer specific or verifiable details of restaurant numbers or percentages that remained opened for dine in options, remained open for takeaway or delivery options, or closed completely.

Because of this inability to find supporting evidence on availability, we slightly shifted the language and logic of this mechanism. Rather than describe a mechanism based on decreased restaurant availability, we decided to describe a mechanism based on decreased restaurant sales. Moreover, we believe one of the best sources for this information is our Reference source [1], which claims that "restaurant spending declined by approximately one-third" during the school closure period of the pandemic. We hope that this somewhat subtle shift in language and logic will better resonate with Reviewer 2 and general readers.

Why restrict the time period? Is it possible to extend the comparison and keep the control category (i.e. correct reference month before the pandemic) the same?

We agree that it would be useful and interesting to extend the time frame. However, doing so would require us to change our identification strategy, and we are reluctant to do that. To explain, recall that our monthly food purchase observations immediately before and during the pandemic are being compared to the monthly food purchases from the exact same households one year prior. Thus, one household's purchases for the 4-week period following school closures in that household's state (say March 15-April 11, 2020, for a Michigan household) are compared to the same household's purchases 52-weeks earlier (say, March 17-April 13, 2019). This strategy essentially controls for household-level idiosyncrasies and seasonality. Lastly, recall that we track purchases 6 months prior to the school closures to 6 months after school closures, for a full 12 months.

Thus, if we extended the time frame to include additional months after school closures, our one-year prior reference group strategy would no longer work, since the reference group timing would encroach on the pandemic period.

The bottom line is that we believe that benefits our retaining current identification strategy, which relies on the household-specific purchases one year prior, outweighs the "cost" of restricting the time period.

All of the figures need vertical axis labels.

Agreed. We have made this fix.

In Table 2, is it possible to calculate the mean scores by month for control and pandemic? This might help provide additional intuition to support the regression estimates especially since the differences between annual averages don't look meaningful. And by month I mean the recoded 4 week periods constructed in the analysis, not necessarily calendar months.

Yes, this additional month-by-month information sounds like a good idea. We have added a brand new table (Table 3) that summarizes this information, as well as accompanying text to briefly describe the table.

It would be helpful if the article could do more to discuss the real significance of the measured change. For example, what might a 2 percent change in the Berry Index look like? Is that just consumption of one more product or a fraction of a product?

This question of what a 2 percent increase in the *Berry Index* looks like, and the related question of what its real significance is sounds simple, but in fact quite complicated. Since the *Berry Index*, as we construct it, is based on expenditure shares (rather than quantities) from 24 food groups, a 2 percent increase might arise from a large change in

the food group with the highest or lowest share, or from a small change in all food group shares.

To help better answer this question, we conducted some hypothetical calculations of the *Berry Index*. First, we found that the *Berry Index* would increase 2.1% if a household moved from consuming equal shares of 7 categories to equal shares of 8 categories. Second, using the data from our full sample, we calculate that a 2% increase in the B*erry Index* from the sample mean increases the *Berry Index* by 0.0165, and that this level of increase is about the same magnitude as a household moving from the 75th percentile of the *Berry Index* to the 90th percentile.

We acknowledge that these calculations really may not help readers very much, so we are currently choosing not to add more explanation to the revised manuscript. If Reviewer 2 thinks they are helpful, then we will be glad to add them to the manuscript.

More generally, we feel that Reviewer 2's next question might be more fruitful to pursue.

Relatedly, from construction of the indices can the authors assess where the increased diversity came from? Even just looking at the broad categories might be instructive. For example, given the supply and income shocks motivating the manuscript we might think diversity declined due to stock outs and limited income. However, given the finding of increasing diversity were consumers purchasing additional fruits/vegetables or something else?

This question, which asks about what's happening "inside" the *Berry Index* (and perhaps also the *USDAScore*), is a completely valid and interesting questions. However, it's a hard question to answer given the large number of food categories and heterogeneity across households. That said the next two tables -- Reviewer Report (RR) Tables 1 and 2 might help with this answer.

RR Table 1: Difference in average of year-on-year percentage changes in spending between pre-school closure months (-6≤m≤-1) and post-school closure months (1≤m≤6)

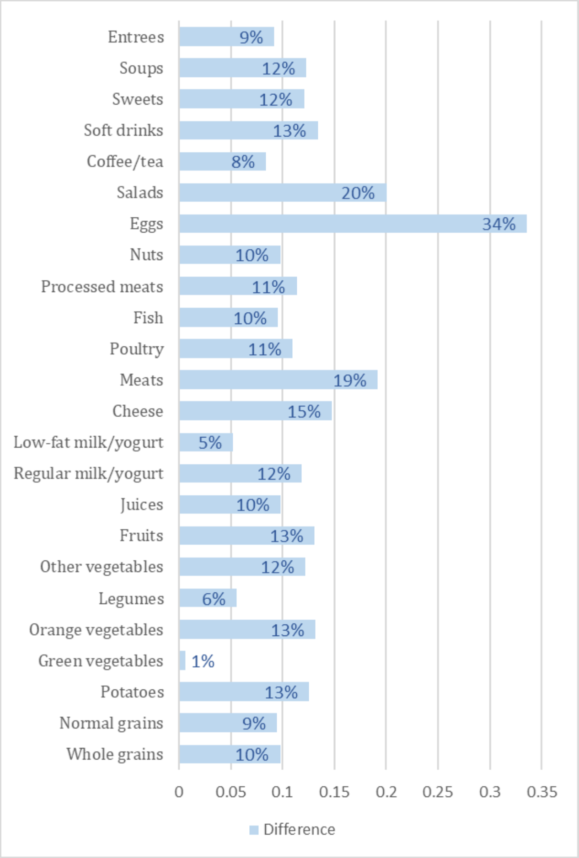

RR Table 1 shows that spending on all CNPP food categories increased, on average, during the school-closure period, though some increased more than others. This increased spending is likely caused by (a) expenditure shift from FAFH to food at home and (b) higher prices for some food categories. For example, the egg and meats categories, two categories known to have higher prices after COVID, have some of the largest spending increases.

That said, since both the *Berry Index* and *USDAScore* are based on expenditure shares rather than expenditure levels, looking inside these scores requires one to consider the relative change in total food spending. RR Table 2 helps with this task and shows how expenditure shares for the CNPP categories changed in the school closure period. The eggs category still has the highest relative increase in spending, but now we can also see salads and meats also had higher shares during the school-closure period. The categories that experienced decreased shares vary widely and include both healthy categories (e.g., -13% for green vegetables, -7% for legumes, -5% for whole grains) and unhealthy categories (e.g., -4% for entrees, -4% for normal grains, and -2% for sweets).

RR Table 2: Difference in average of CNPP category expenditure shares between pre-school closure months (-6≤m≤-1) and post-school closure months (1≤m≤6)

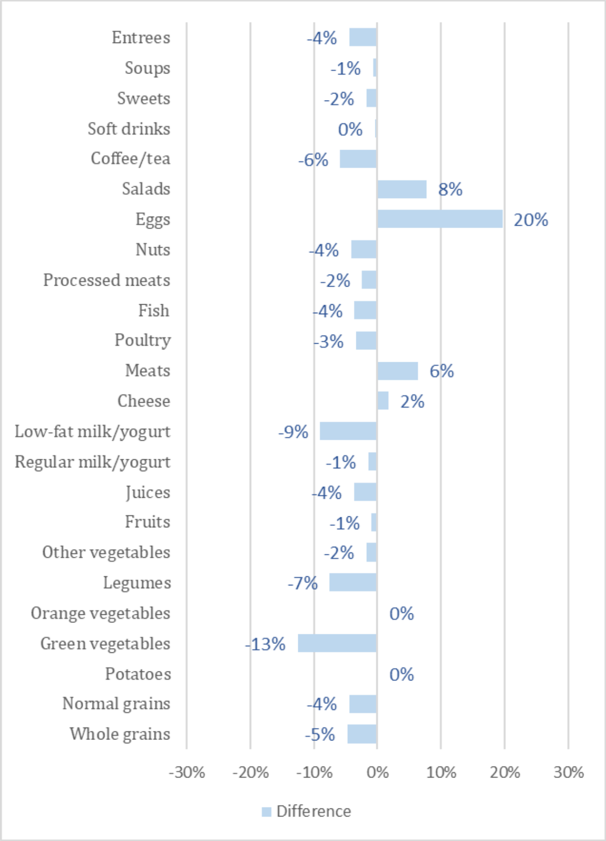

However, even the share changes in RR Table 2 mask some of the "action" inside the *Berry Index*. To better understand the *Berry Index* changes, one would need both the information in RR Table 2 and the "starting points" that show just how uneven the shares are, and which are large and which are small. Our original Table 1 can help with this task. For example, the eggs category in Table 1 has one of the smallest actual monthly expenditure shares (0.97%), so the large increase in the egg share (as seen in RR Table 2), means that this increase will help make the shares "more even" and raise the *Berry Index*. Additionally, (regular) whole milk and yogurt had a high share in Table 1, so the slight decrease in this share (as seen in RR Table 2), also leads to a higher *Berry Index*, all else equal.

These additional tables are meant to highlight the notion that there are no easy answers to Reviewer 2's question. Additionally, we currently do not think it would be helpful to readers to add these tables (and explanation) to the manuscript. If Reviewer 2 feels that this information would be helpful, we will gladly add it.

More details on the construction of the alternate demographic variables would be helpful. For example, what income levels were considered? Why is single income grouped with no income?

Summary statistics for the sample and definitions for what the actual survey question looked like would be helpful.

We added a new appendix table (S4) that explains the definition or construction of the demographic variables and lists basis summary statistics. In addition, as Review 3 suggests, we now split the income source group into three: households where no household head earns income, households that have a single household head earning income, and households that have two household heads earning income.

In addition, we made changes to the text that explains Fig 2. Namely, we described one observation for each of the six panels; we used the (a) through (f) labeling from Figure 2 in the text so readers could better match our explanation to the figure, and finally we added in variable definitions to our explanations.

Are the authors able to add any discussion of income effects that may have also been at play during the COVID-19 pandemic? This is especially important given the great deal of research supporting dietary diversity changes with income. Also it appears there is a sharper decline for the low income category in Figure 2 in the months following the pandemic.

The revised manuscript does now mention the sharper decline in the *Berry Index* seen in Figure 2. So now, we have short passages that note income differences in how the *Berry Index* and the *USDAScore* change after the pandemic.

However, we are a bit reluctant to say more about the overall income effect, mainly because there are important things that are unobservable in the data. For example, we can't observe which households experienced a job loss or reduction of hours worked during the pandemic. Nor do we observe which household heads were able to continue working remotely and avoid the costs of commuting. Finally, we also do not observe which households received pandemic relief payments from the government or other forms of pandemic assistance.

Response to Reviewer 3

Please refer to the introductory section of this report to read how appreciative we are for the reviewers' comments and to see a summary of our major revisions. The following section addresses Reviewer 3's comments one by one.

1. *My biggest concern is that the study uses the food purchase scanner data to measure consumers' diet quality and diversity. I think such measurements may not necessarily accurately reflect consumers' diet habits. Multiple studies have documented the stockpiling food purchasing behavior immediately after the pandemic (e.g., Amaral et al., 2022; El Baba & Fakih, 2023), so I suspect that the increase in Berry Index and USDAScore could also be caused by consumers' stockpiling food purchasing behavior instead of their food consumption behavior. In addition, food prices experienced changes after the pandemic, so using the expenditure on food products may not be an accurate measurement of consumers' food consumption behavior.*

Thank you for raising these concerns, which we had considered but did not address in the original manuscript. As a result of this comment, we added a third set of caveats in our Discussion section. More specifically, we discuss stockpiling (and add the two cited papers), but we ultimately say that we hope our study period, which lasts six months after school closures, mitigates concerns that stockpiling at the outset of the closure period might affect the two diet quality measures. We also discuss food inflation and how it may or may not affect the Berry Index. In short, we say that since the *Berry Index* is based on expenditure shares (rather than expenditure levels), and since food inflation could artificially increase or decrease the *Berry Index* depending on how large or small a household's shares are, we again hope that our results are not overly affected by these concerns.

Amaral, N. B., Chang, B., & Burns, R. (2022). Understanding consumer stockpiling: Insights provided during the COVID-19 pandemic. *Journal of Consumer Affairs*, *56*(1), 211-236.

El Baba, W., & Fakih, A. (2023). COVID-19 and consumer behavior: Food stockpiling in the US market. *Agribusiness*, *39*(2), 515-534.

2. *Page 6 lines 116-117: Equation (1) has multiple mistakes. First, it is missing subscript for variables such as $PandemicYear$. Also, the equation misses the fixed effect $PandemicYear_i$. The two-way fixed effect (WTFE) regression model needs to include both treatment group and time period variables in the regression (see S1 Table, which did include the $PandemicYear_{i,y}$ variable in the regression).*

Thank you for catching these errors. The revised Equation (1) now has an *i* subscript on $PandemicYear$ and the missing term, + $\gamma PandemicYear_i$. However, we use only the *i*

subscript on this new term since it takes only two values, 0 and 1, and we omit the base case when its equal to 0. We think this notation makes the most sense. However, if the reviewer feels strongly that we should add the y subscript, for consistency's sake, we will add it.

3. *Page 7 line 137: Please consider change the subscripts in Equation (2). You have used the subscript* i *to indicate the household in equation (1). Using the subscript* i *in equation (2) is misleading. Also, in Equation (3), you have the subscript* imy *for USDAScore, please consider also using the same subscript for Berry Index (BI).*

   Again, thanks for catching these errors and inconsistencies. We have fixed them along the lines that you suggest.

4. *Page 9 line 166: I do not think the USDAScore in Equation (3) should include the subscript* c *because there should not be a USDAScore for each food category.*

   Same as above. We fixed this as you suggest. Thanks.
