# Supplementary material for: Pandemic-induced changes in household-level food diversity and diet quality in the U.S
Source: PLoS One. 2024 May 31;19(5):e0300839. doi: 10.1371/journal.pone.0300839 (PMC11142597; doi:10.1371/journal.pone.0300839)
Supplement: Attachment — Submitted filename: PLOS one review 12 Oct 23.docx [file pone.0300839.s005.docx]

This was an interesting manuscript which examines changes in household food purchasing practices six months before and after the COVID-19 pandemic in the US.

The approach to assessing diet quality using two indicators seemed sound, and the interpretation of findings was appropriate.

I do not have expertise in the modelling and regression techniques used in the methods and results and have therefore not commented on these aspects.

**General comments and suggested revisions**

The abstract would benefit from providing more summary information about the study (specific suggestions are detailed below).

The introduction is rather brief and could include more of the literature on dietary changes during the pandemic. Where individual studies are referred to from a secondary source (e.g. a systematic review), the original study should be cited/referenced.

There was inconsistent use of past and present tense throughout the manuscript. Past tense throughout would be preferable.

Discussion Lines 289 Some households (those where job security or household income was not affected) may have had more disposable income due to savings in costs for transportation, childcare and recreation. Would greater disposable income contribute to selection of more healthful food choices since these are sometimes more costly relative to foods high in energy and low in nutrients?

**Specific comments**

Abstract

Add time points (year/month) for pre-pandemic measures vs pandemic measures of diet quality. If this is not possible because of the varying timing across states, suggest adding text along the lines of what is written in the methods e.g. ‘from 6 months before to 6 months after the event month’.. of school-closure in each state in response to the pandemic.

Provide an indication of sample size/number households from which data were obtained.

Lines 19 & 20 Clarify whether percentages are ‘percent’ or ‘percentage points’.

State what statistical analyses were performed.

Lines 307-309 of the conclusion would be relevant to include in the abstract.

Introduction

Line 26, for context it would be helpful to add when (year & month) the majority of states closed restaurants and cafeterias. L

Line 39-40 ‘the pandemic-induced closing of dine-in restaurants’ presumably takeaway restaurants and food outlets remained open throughout? This could be stated to make it clear that potentially ‘unhealthy’ or ready-to-eat foods were still available, assuming this was the case in most states.

Line 49 – it would be helpful to add how diet quality was measured in the study reference [9], and which diet quality measures were used for the four US studies.

Line 49- add the citations for all four of the US studies i.e. add the remaining two in addition to ref [10,11].

Line 59 could ‘expenditure shares’ be explained in simple terms on first use, e.g. ‘share spent on a food category out of the total household purchase expenditure'?

Line 58 to 79 – for articles on nutrition, these details on the construction of a dietary index may fit better in the methods section rather than the introduction.

Materials and methods

Line 85 – ‘data’ is usually written in the plural, ie. ‘these data allow the study’ instead of ‘this data’

Line 145-146 -Is the share based on total household purchase expenditures, including non-food items, and how might this have changed during the pandemic (i.e. purchasing more non-food items from a single retail outlet to avoid multiple contacts/exposures)?

Line 147: Has CNPP been defined in full previously? Same for the Table 1 heading.

Table 1: I wasn’t clear where the ‘Average actual monthly expenditure’ share comes from in the column heading. For what year, or sample- is this the same 80,000 households over the full data period before dividing into pre-and post pandemic? Please ensure that Table 1 can be fully understood without needing additional information from the manuscript.

Line 197- is there a word missing in ‘each household’s monthly score their score’?

Line 217 and 230 Change ‘white households’ and ‘Asian households’ to ‘Households classified as white ethnicity…etc

Line 240 – show the evidence that food purchasing behaviour of US household was dramatically affected. I couldn’t see that this paper has shown dramatic changes in behaviour per se, but they may have changed food expenditure patterns. It would be incorrect to assume that this led directly to changes in food consumption patterns, since this has not been assessed.

Figures 2 and 3: Is ‘race’ the appropriate term for figure headings (d)? Conventions vary in different global regions, ‘ethnicity’ may be an alternative term to use. The journal may give some guidance on this.
