# Supplementary material for: Pandemic-induced changes in household-level food diversity and diet quality in the U.S
Source: PLoS One. 2024 May 31;19(5):e0300839. doi: 10.1371/journal.pone.0300839 (PMC11142597; doi:10.1371/journal.pone.0300839)
Supplement: Attachment — Submitted filename: review_report.docx [file pone.0300839.s007.docx]

This paper employs Different-in-Difference (DiD) methods to investigate the impact of the Covid-19 pandemic on US consumers’ diet diversity and diet quality. Using the Neilson food purchasing scanner data, the study finds that the pandemic increases both food diversity (indicated by Berry Index) and diet quality (indicated by USDAScore). However, the magnitude of the increase becomes smaller as the time period is further away from the date that schools shut down because of the pandemic.

In general, I find this paper to be well-written and consider its research topic to be of significant importance to the field. Understanding consumers’ diet change caused by the pandemic holds substantial implications for developing food policy to increase US consumers’ healthy food consumption. Nevertheless, I have some concerns in this paper, which I have detailed below.

1. My biggest concern is that the study uses the food purchase scanner data to measure consumers’ diet quality and diversity. I think such measurements may not necessarily accurately reflect consumers’ diet habits. Multiple studies have documented the stockpiling food purchasing behavior immediately after the pandemic (e.g., Amaral et al., 2022; El Baba & Fakih, 2023), so I suspect that the increase in Berry Index and USDAScore could also be caused by consumers’ stockpiling food purchasing behavior instead of their food consumption behavior. In addition, food prices experienced changes after the pandemic, so using the expenditure on food products may not be an accurate measurement of consumers’ food consumption behavior.
2. Page 6 lines 116-117: Equation (1) has multiple mistakes. First, it is missing subscript for variables such as $PandemicYear$. Also, the equation misses the fixed effect $PandemicYear_{i}$. The two-way fixed effect (WTFE) regression model needs to include both treatment group and time period variables in the regression (see S1 Table, which did include the $PandemicYear_{i, y}$ variable in the regression).
3. Page 7 line 137: Please consider change the subscripts in Equation (2). You have used the subscript $i$ to indicate the household in equation (1). Using the subscript $i$ in equation (2) is misleading. Also, in Equation (3), you have the subscript $imy$ for *USDAScore*, please consider also using the same subscript for Berry Index (BI).
4. Page 9 line 166: I do not think the *USDAScore* in Equation (3) should include the subscript $c$ because there should not be a *USDAScore* for each food category.

References

Amaral, N. B., Chang, B., & Burns, R. (2022). Understanding consumer stockpiling: Insights provided during the COVID‐19 pandemic. *Journal of Consumer Affairs*, *56*(1), 211-236.

El Baba, W., & Fakih, A. (2023). COVID‐19 and consumer behavior: Food stockpiling in the US market. *Agribusiness*, *39*(2), 515-534.
