# Supplementary material for: Pandemic-induced changes in household-level food diversity and diet quality in the U.S
Source: PLoS One. 2024 May 31;19(5):e0300839. doi: 10.1371/journal.pone.0300839 (PMC11142597; doi:10.1371/journal.pone.0300839)
Supplement: Attachment — Submitted filename: Referee report for PONE-D-23-26121.docx [file pone.0300839.s006.docx]

Referee report for: PONE-D-23-26121

Summary: This manuscript assesses changes in household dietary diversity using two different measures during the year following the COVID-19 pandemic. The manuscript uses an event study design with a pre-pandemic control group constructed based on the beginning of school lockdowns at the state level. Additionally, the study uses scanner data to construct the dietary diversity measures. Overall, the study finds that the COVID-19 lockdowns were associated with increases in the dietary diversity score (across both measures). The manuscripts proceed to discuss several mechanisms for this increase including the decreased availability of food away from home, changes in health consciousness, supply chain stockouts, and changing time constraints.

- In the conclusion and discussion section, the proposed mechanism of decreased FAFH availability seems outside the scope of this study. As correctly noted, the present measures do not include any FAFH purchases and the study provides no evidence on the prevalence of restaurant closures or FAFH availability during these periods across states. Anecdotally, FAFH available for takeout and delivery appears to have been widely available during this period. Also, pre-prepared foods at grocery retailers may have similar health attributes as FAFH and could have been included in these grocery baskets. Again, I think this mechanism is somewhat of a stretch given the limits of the data presented in this article.
- Why restrict the time period? Is it possible to extend the comparison and keep the control category (i.e. correct reference month before the pandemic) the same?
- All of the figures need vertical axis labels.
- In Table 2, is it possible to calculate the mean scores by month for control and pandemic? This might help provide additional intuition to support the regression estimates especially since the differences between annual averages don’t look meaningful. And by month I mean the recoded 4 week periods constructed in the analysis, not necessarily calendar months.
- It would be helpful if the article could do more to discuss the real significance of the measured change. For example, what might a 2 percent change in the Berry Index look like? Is that just consumption of one more product or a fraction of a product?
- Relatedly, from construction of the indices can the authors assess where the increased diversity came from? Even just looking at the broad categories might be instructive. For example, given the supply and income shocks motivating the manuscript we might think diversity declined due to stock outs and limited income. However, given the finding of increasing diversity were consumers purchasing additional fruits/vegetables or something else?
- More details on the construction of the alternate demographic variables would be helpful. For example, what income levels were considered? Why is single income grouped with no income? Summary statistics for the sample and definitions for what the actual survey question looked like would be helpful.
- Are the authors able to add any discussion of income effects that may have also been at play during the COVID-19 pandemic? This is especially important given the great deal of research supporting dietary diversity changes with income. Also it appears there is a sharper decline for the low income category in Figure 2 in the months following the pandemic.

The manuscript references supply chain shocks, but at the same time we know there were income shocks which also occurred both from lost employment and from government distribution of payments. For example, Lai et al. (2020) find that over 65% of their sample had received an Economic Impact Payment by mid-May 2020 which covers the early period of most of your sample and may have influenced purchasing or consumption behavior. Below are two references which I think may be helpful at framing this discussion that look at consumer purchasing and expenditures: Bina et al. (2023) and Lai et al. (2020).

**References:**

Bina, J. D., Tonsor, G. T., & Briggeman, B. C. (2023). COVID-19 Federal Aid and Household Food Expenditures. *Journal of Agricultural and Applied Economics*, *55*(4), 567-608.

Lai, J., Morgan, S., Kassas, B., Kropp, J., & Gao, Z. (2020). Spending of economic stimulus payments and changes in food purchasing during the COVID-19 pandemic. *Choices*, *35*(3), 1-8.
